# High‐Fidelity Computational Microscopy via Feature‐Domain Phase Retrieval

**DOI:** 10.1002/advs.202413975

**Published:** 2025-02-22

**Authors:** Shuhe Zhang, An Pan, Hongbo Sun, Yidong Tan, Liangcai Cao

**Affiliations:** ^1^ Department of Precision Instruments Tsinghua University Beijing 100084 China; ^2^ State Key Laboratory of Transient Optics and Photonics Xi'an Institute of Optics and Precision Mechanics Chinese Academy of Sciences Xi'an 710119 China; ^3^ University of Chinese Academy of Sciences Beijing 100049 China

**Keywords:** computational imaging, feature domain, microscopy, optimization, phase retrieval

## Abstract

Computational microscopy enhances the space‐bandwidth product and corrects aberrations for high‐fidelity imaging by reconstructing complex optical wavefronts. Phase retrieval, a core technique in computational microscopy, faces challenges maintaining consistency between physical and real‐world imaging formation, as physical models idealize real phenomena. The discrepancy between ideal and actual imaging formation limits the application of computational microscopy especially in non‐ideal situations. Here, the feature‐domain consistency for achieving high‐fidelity computational microscopy is introduced. Feature‐domain consistency tells that certain features, such as edges, textures, or patterns of an image, remain invariant in different image transformations, degradations, or representations. Leveraging the feature‐domain consistency, Feature‐Domain Phase Retrieval (FD‐PR) is proposed, a framework applicable to various computational microscopy. Instead of working directly with images' pixel values, FD‐PR uses image features to guide the reconstruction of optical wavefronts and takes advantage of invariance components of images against mismatches of physical models. Experimental studies, across diverse phase retrieval microscopic tasks, including coded/Fourier ptychography, inline holography, and aberration correction, demonstrate that FD‐PR improves resolution by a factor of 1.5 and reduces noise levels by a factor of 2. The proposed framework can immediately benefit a wide range of computational microscopies, such as X‐ray ptychography, diffraction tomography, and wavefront shaping.

## Graphical abstract

An innovative phase retrieval framework, termed FD‐PR, is uniquely established in the image's feature domain through the feature‐extracted, physical‐driven regression with interfaces for combining physics and image processing constraints. FD‐PR takes advantage of invariance components of an image against presences of model mismatch and uncertainty, which can immediately benefit a wide range of computational microscopies in practical, non‐ideal conditions.

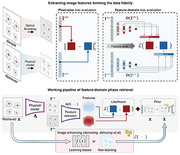

## 1 Introduction

Microscopy enables the study of structures too small to be seen by the naked eye, playing a crucial role in fields like biological and medical research,^[^ ^1^ ^]^ materials science,^[^ ^2^ ^]^ pharmaceutical development,^[^ ^3^ ^]^ as well as environmental science.^[^ ^4^ ^]^ However, current optical detectors capture only intensity, omitting phase information, which is vital for fully characterizing optical phenomena.^[^ ^5^, ^6^ ^]^ Phase retrieval addresses the challenge by computationally recovering the complex amplitude of an optical wavefront from intensity‐only measurements without interferometric optical settings.^[^ ^7^ ^]^ Reconstructed wavefronts reveal phase and non‐diffracted amplitude, enabling super‐resolution techniques like Fourier ptychography,^[^ ^8^, ^9^ ^]^ quantitative phase fluorescence imaging,^[^ ^10^, ^11^ ^]^ and X‐ray and electron ptychography.^[^ ^12^, ^13^ ^]^ Additionally, phase retrieval compensates for aberrations,^[^ ^14^, ^15^ ^]^ and facilitates computational tomography^[^ ^16^, ^17^, ^18^ ^]^ to retrieve the 3D refractive index of samples when combined with 3D scattering theory. Furthermore, by designing specific phase or amplitude patterns, phase retrieval enables the creation of unusual light structures,^[^ ^19^, ^20^ ^]^ expanding research into object‐wave interactions.^[^ ^21^ ^]^

Phase retrieval is a regressive task, where the goal is to estimate the target wavefront that best fits the physical model, defined by the image formation process, and reproduces the intensity‐only measurement.^[^ ^22^ ^]^ The success of phase retrieval relies heavily on the consistency between the predefined physical model and real‐world image formation. However, ensuring consistency is challenging due to the simplifications inherent in physics modeling,^[^ ^23^ ^]^ particularly in non‐ideal imaging scenarios. To enhance phase retrieval quality without altering the optical setup, algorithmic advancements play significant roles.^[^ ^23^, ^24^, ^25^ ^]^ Conventional methods, categories from alternative projection methods,^[^ ^26^, ^27^, ^28^ ^]^ to more recent non‐convex optimization methods,^[^ ^29^, ^30^, ^31^ ^]^ are established based on complex least‐square regression (CLSR). CLSR solves phase retrieval by minimizing the pixel‐wise difference between experimental and model‐predicted images. Despite its efficiency via alternative projection and compatibility with convex optimization tools like proximal gradients,^[^ ^32^ ^]^ CLSR struggles with system outliers such as parameter mismatch^[^ ^33^, ^34^ ^]^ or non‐linearity.^[^ ^35^ ^]^ Other approaches, including Poisson^[^ ^36^ ^]^ and Gaussian–Poisson^[^ ^37^ ^]^ likelihoods, have been explored but still simplify real‐world noise. Deep‐learning methods^[^ ^38^, ^39^, ^40^ ^]^ show promise for specific tasks but lack generalizability across diverse problems. These challenges underscore the need for more robust, non‐convex optimization techniques in phase retrieval.

Despite the generalization challenges of deep‐learning methods, their success in pattern recognition and image restoration under challenging conditions suggests untapped potential.^[^ ^41^, ^42^ ^]^ Central to deep learning's effectiveness is the concept of feature‐domain consistency, which refers to the invariance of certain image features, such as edges and textures, across degradations and transformations like scaling or rotation.^[^ ^43^, ^44^ ^]^ In image restoration, feature‐domain consistency enables deep neural networks to extract multi‐level representations, ensuring high fidelity.^[^ ^45^ ^]^ CLSR cannot capture perceptual differences between model‐predicted and observed intensities, relying heavily on high‐quality imaging and accurate physical models.^[^ ^46^ ^]^ For example, two identical images offset by one pixel may appear perceptually similar but exhibit substantial pixel‐wise differences. CLSR attempts to address such offsets by incorporating translation matrices into image formation models. However, this approach becomes inefficient in complex scenarios with high uncertainty.

In this research, we report the feature‐domain phase retrieval (FD‐PR) for achieving high‐fidelity computational microscopy. The framework stands apart from off‐the‐shelf image‐domain phase retrieval methods that are based on image‐domain CLSR.^[^ ^26^, ^29^, ^30^, ^31^, ^47^ ^]^ Instead of working directly with pixel values as in the image domain, FD‐PR uses feature‐domain consistency and focuses on ensuring that important image features extracted from a learned or predefined feature extractors remain stable, even when the image undergoes noise, blur, downsampling, or other modifications. The regression of FD‐PR is uniquely established in the image's feature domain through the feature‐extracted non‐linear forward model with intensity scaling functions. FD‐PR is further designed with interfaces for arbitrary constraints, refining the recovered wavefront during optimization. Given the non‐convex, non‐linear nature of the loss function, FD‐PR resembles the training of a deep neural network, where the target wavefront is learned by minimizing the loss function through back‐propagation. FD‐PR can handle intricate, differentiable feature‐domain likelihood functions, and leverages advanced optimization and learning strategies from deep learning for effective phase retrieval.

## 2 Results

### 2.1 The Pipeline for Feature‐Domain Phase Retrieval

**Figure 1:**
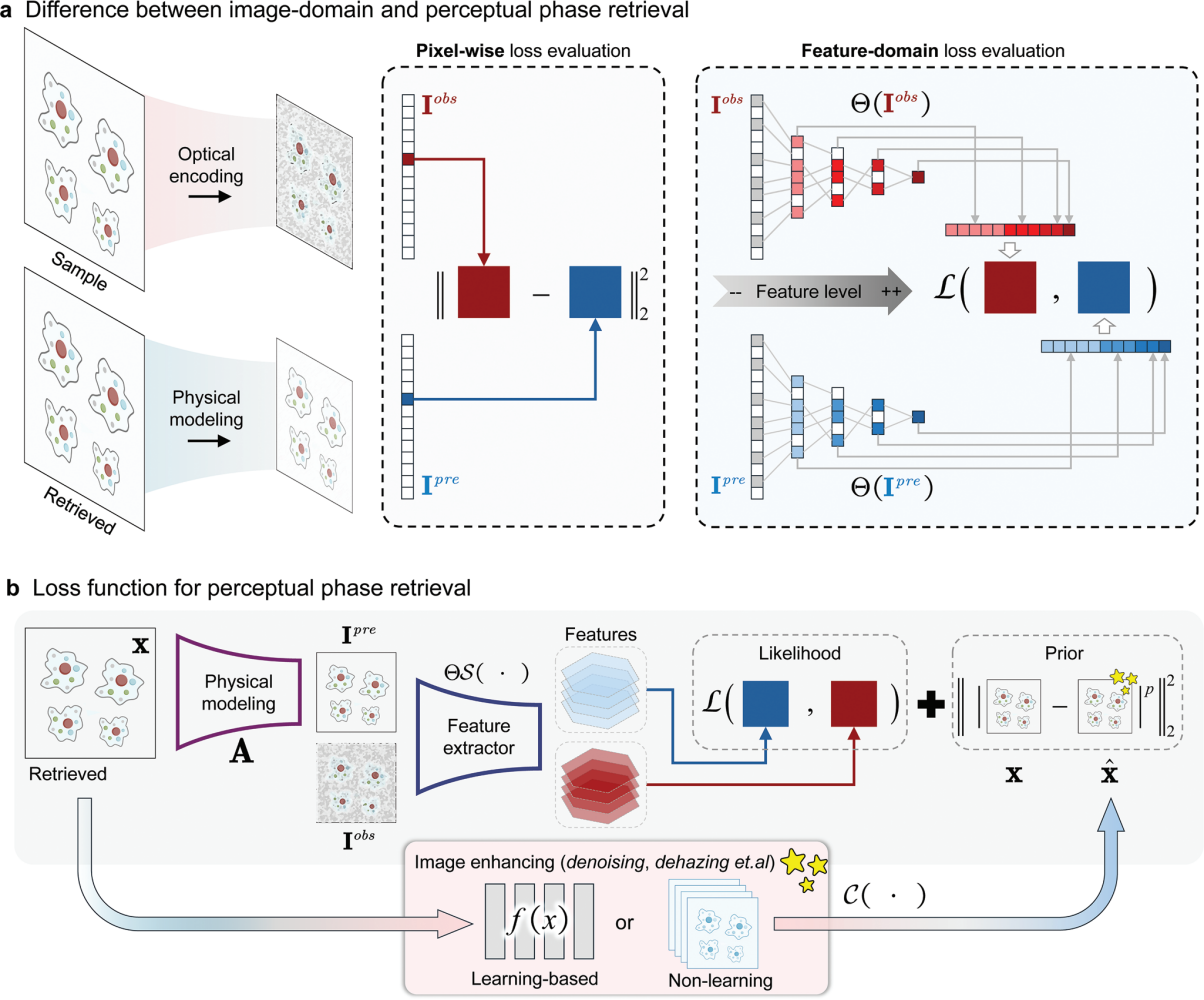
The framework of FD‐PR with feature‐domain likelihood. a) Concept of using image's features for loss function compared to conventional pixel‐wise difference loss function. b) The flow chart of FD‐PR. FD‐PR is based on the unified linear image formation model. FD‐PR treats phase retrieval tasks as regressions where the latent wavefront can be learned from the measured intensity through gradient descent. The observed intensity is fed to FD‐PR, and the parameters are learned through complex back‐propagation accelerated by given optimizers.

The idea of FD‐PR is depicted in **Figure** **1**a. As a regressive task, the target wavefront, denoted by **x**, is recovered by minimizing the difference between model predictions and experimental observations. Conventional phase retrieval uses the pixel‐wise difference to guide the regression. Instead of working directly with pixel values as in the spatial domain, FD‐PR focuses on the image's features extracted from a learned or predefined feature extractor, using the feature‐domain difference to guide the regression. The feature‐domain loss function for FD‐PR is given as.

(1) $$\begin{array}{cc} L_{\text{FD-PR}}\left(x,A_{k}\right) & =\sum_{n=1}^{N}D\left[\Theta S\left(I_{n}^{obs}\right),\;\Theta S({\left|A_{n}x\right|}^{2})\right]\;\;\;\;\\ & \;+\alpha {\left\|{\left|x-C(x)\right|}^{p}\right\|}_{2}^{2}+\sum_{k=1}^{K}\beta_{k}\;{\left\|{\left|A_{k}-C\left(A_{k}\right)\right|}^{p}\right\|}_{2}^{2}\;\;\;\; \end{array}$$

where D evaluates the distance between intensity observation and model prediction. **Θ** is the feature extractor and S is the intensity scaling function. **x** is the input of the optical system described by propagator **A**. C denotes arbitrary constrain functions such as denoising or dehazing for image enhancement. Notes S1 (Supporting Information) provides necessary details for completing the feature‐domain likelihood.

**A**_*n*_ in Equation (1) denotes the *n*‐th linear propagator of an optical system for *n*‐th intensity observation $I_{n}^{obs}$. **A**_*n*_ can be further separated into several linear operators given as

(2) $$A_{n}=A_{k}A_{K-1}\cdots A_{k}\cdots A_{2}A_{1}=\prod_{k=K}^{1}A_{k}$$

The goal of phase retrieval is to recover **x** and possibly unknown parameters in $A_{k}$ in Equation (2) from intensity observation. α and β are penalty parameters.

Figure 1b shows the framework for FD‐PR. The loss function comprises two blocks, and the first is the feature‐domain augmented likelihood block that uniquely maximizes the data likelihood in the image's feature‐domain, in contrast to the image‐domain likelihood widely used in conventional phase retrieval methods. The feature‐domain likelihood is the core of FD‐PR, which is established on the image's feature extracted by invertible feature‐extracting operators. The second block is the constraint block which implements our proposed extended‐HIO (eHIO), providing plug‐and‐play interfaces for arbitrary customized constraints including thresholding constraints, image processing constraints, and physical constraints, during the non‐convex optimization routine. As shown in Figure 1b the complex gradient given by the likelihood block and constraints block with a certain image post‐processing refinement is calculated from a current estimation of model parameters. Please refer Note S2 (Supporting Information) for the eHIO block.

### 2.2 FD‐PR for Stitching‐Free Full‐Field Fourier Ptychography

**Figure 2:**
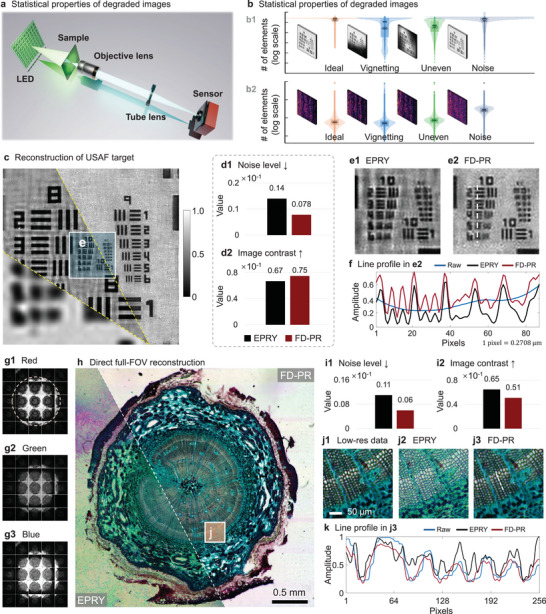
FPM reconstruction from feature‐domain extracted by Bior 2.2 wavelet. a) Sketch of FPM platform. b) The statistical analysis of pixel value possibility of given images under different degrading conditions in b1) image domain and b2) feature domain. c) Reconstruction result for USAF resolution target using EPRY^[^ ^49^ ^]^ and FD‐PR. d1,d2) Image quality evaluation using noise level measurement and image contrast measurement for image (c). e1,e2) Zoomed‐in image in the area marked by the blue box in (c). f) Amplitude profiles along the line in (e2). g1–3) First 25 images for red, green, and blue LED illuminations. The central wavelengths for the red, green, and blue LEDs are 0.683 μm, 0.532 μm, and 0.488 μm, respectively. h) Montage of direct, 16K, full‐FOV reconstruction of pine stem sample using FD‐PR and EPRY. i1,2) Image quality evaluation of (h) using noise level and image contrast. j1–j3) Zoomed‐in images for the area marked by the orange box in (h). k) Line profile along the white dashed line in (j3).

We conducted a Fourier ptychographic microscopy (FPM) as shown in **Figure** **2**a for pine stem sample and USAF resolution testing target. Experimental parameters can be found in Note S4 (Supporting Information). The vignetting effect is a common and challenging outlier in FPM,^[^ ^35^ ^]^ which can only be solved by modifying the optical system in the conventional FPM routine.^[^ ^48^ ^]^ In the presence of outliers to the raw image, direct image‐domain phase retrieval causes unexpected artifacts and decreases the recovery quality, especially when the input images have sample‐induced intensity fluctuations or vignetting effects. FD‐PR utilizes the feature extracting **Θ** to form the likelihood term that can bypass the impact of some image domain degrading giving high robustness to feature‐unrelated outliers. As features are inherent properties of images, feature‐domain consistency ensures that features are more robust to diverse kinds of degrading than the image itself.

To show the feature‐domain consistency, we use the bi‐orthogonal 2.2 (Bior 2.2) wavelet decomposition, denoted by **Θ**, to extract five levels of image features. Figure 2b shows images that are subject to various forms of image degrading, such as uneven background and noise signals. Each of these factors introduces unique statistical patterns to the intensity of the image, as illustrated in Figure 2b1 in the image domain. Since the statistical properties are different, it is hard to build a unified model to describe all these degrading situations, posing challenges in maximizing the likelihood between data of different statistical properties. However, when the degraded images are transformed into the feature domain, the statistical patterns of features are reshaped and standardized as shown in Figure 2b2 despite the differences in degrading. Consequently, measuring likelihood in the feature domain is efficient and enhances the robustness of FD‐PR to image‐domain challenges, as well as helps bypass the challenges in the image domain.

We benchmarked the conventional FPM using a widely adopted EPRY^[^ ^49^ ^]^ algorithm, namely the embedded pupil recovery. Experimental results on USAF resolution testing target proved the background‐rectified ability of FD‐PR as shown in Figure 2c. The background recovery is inadequate for EPRY due to uneven illumination intensity and vignetting effect. We further used the noise level and image contrast to evaluate the image quality. Quantitative results are shown in Figure 2d1,d2. FD‐PR achieves a lower noise level in the reconstructed image compared to EPRY, while providing higher image contrast. For a more detailed view, Figure 2e1,e2 shows a zoomed‐in image for the area marked by the blue box in Figure 2c, where FD‐PR obtained a noise‐suppressed reconstructed result with a uniform background. The quantitative plot for USAF's amplitude profile along the white dashed line in **e2** is shown in Figure 2f. The curve plot indicates that FD‐PR obtains higher reconstructed resolution than EPRY, as group 10 element 4 (line width is 0.345 μm) can be resolved, and group 10 element 5 (line width is 0.308 μm) can be almost resolved. Note that the highest resolution achieved by the FPM setup is 0.302 μm @ 0.532 μm wavelength, with a synthetic NA = 0.88.

Furthermore, reconstructing a large field of view (FOV) remains challenging for EPRY due to the vignetting effect, which disrupts the linear invariance required by the FPM principle. This vignetting becomes pronounced under oblique illumination, where vignetted images exhibit a half‐bright, half‐dark pattern, as shown in Figure 2g1–g3 displaying the first 25 images illuminated by red, green, and blue LEDs in a color FPM dataset. FD‐PR achieves a direct, vignetting‐free FPM reconstruction across the entire FOV (10 mm^2^), as shown in Figure 2h. A quantitative evaluation of the image quality for the reconstruction in Figure 2h is provided in Figure 2i1 (noise level) and i2 (image contrast). As seen in Figure 2i1, the overall noise level of FD‐PR is lower than that of EPRY. While Figure 2i2 shows higher image contrast for EPRY, the zoomed‐in views in Figure 2j1–j3 reveal that the ill‐posed reconstruction in EPRY causes color distortion, which contributes to the increased contrast. In contrast, FD‐PR successfully reconstructs the synthetic aperture, allowing individual cells to be clearly observed in Figure 2j3 compared to j1. The quantitative intensity profile for the pine sample, along the white dashed line, is shown in Figure 2k, demonstrating the noise‐suppressed, resolution‐enhanced reconstruction achieved by FD‐PR.

### 2.3 FD‐PR for Blind Large Aberrations Recovery

**Figure 3:**
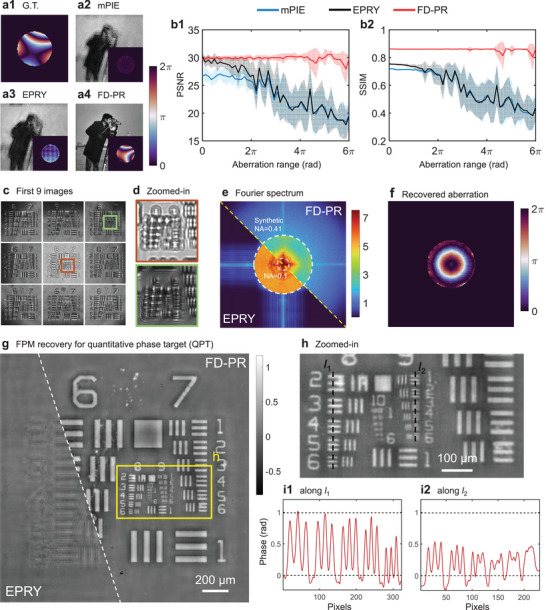
Blind de‐aberration for FPM reconstruction. a1) Ground truth (G.T.) of aberrations for simulation. a2–4) Recovered results for the mPIE, EPRY, and FD‐PR methods. b1,2) The PSNR and SSIM for 500 groups of simulations. Experimental results on the quantitative phase target are shown in (c–g). c) The first 9 images for collected FPM data. d) The zoomed‐in images in the red and green boxes in **(**c). e) The montage of recovered Fourier spectrum of EPRY and FD‐PR methods. f) The recovered aberrations using FD‐PR. g) The recovered phase pattern of EPRY and FD‐PR. The zoomed‐in image for the area in the yellow box is shown in (h). i1,2) The quantitative phase profile along lines *l*_1_ and *l*_2_, respectively.

It is known that conventional FPM cannot handle large aberration data since the EPRY falls into local minima fast and is vulnerable to noise signals and outliers such as vignetting. We demonstrate here using FD‐FPM to recover large aberrations without modifying the optical system through both simulation and experiment. Instead of using wavelet to extract the image's feature, we use the first‐order gradient of the image to guide the regression for both the target sample wavefront and the pupil function. In comparison, FD‐PR outperforms two widely‐used conventional methods, namely the ptychographic iterative engine (PIE) with momentum (mPIE) and the EPRY. The ground‐truth (G.T.) aberration image, depicted in **Figure** **3**a1, exhibits nearly two 2π‐jumps, indicating significant aberration values. Only FD‐PR produces satisfactory reconstruction results, as seen in Figure 3a2 to a4, where sharp images are recovered for FD‐PR.

We conducted extensive simulations, employing the first 15 orders of Zernike functions to generate the phase of the pupil function. Both the peak signal‐to‐noise ratio (PSNR) and the structure similarity index measurement (SSIM) were used to evaluate the image's quality. The results, depicted in Figure 3b1,b2, showcase the robustness of FD‐PR in achieving high‐quality reconstructions across various aberration ranges. While both mPIE and EPRY perform adequately when the aberration is small, their reconstruction quality significantly deteriorates as the aberration increases. This deterioration indicates a failure of FPM reconstruction under these conditions. Please refer Note S4 (Supporting Information) for experimental parameters.

We conduct experimental results for reconstruction over large aberrations. The quantitative phase target is placed on an unknown defocused plane so that the significant diffraction patterns can be observed as shown in Figure 3c for the first 9 images, and Figure 3d for zoomed‐in areas in the red and green boxes. Additionally, the images are corrupted by the vignetting effect, exacerbating the reconstruction challenges. Nevertheless, FD‐PR outputs satisfactory reconstruction results as evidenced in Figure 3e for the sample's Fourier spectrum and Figure 3g for the reconstructed phase pattern. The recovered aberration, shown in Figure 3f, has nearly two 2π‐jumping indicating significant aberration varying from 0 to 4π. Conversely, the reconstruction of EPRY fails without a priori defocus distance for aberration compensation, as the algorithm is stuck into local minima. Using conventional PIE or regularized PIE cannot recover such large aberration as they use quasi‐Newton to determine the step‐size of each iteration, further accelerating the algorithm's fall into local minima. The presence of image‐domain challenges including noise signals, diffractive background intensity, and vignetting effect make image‐domain optimization more challenging. Figure 3h shows the zoomed‐in area in the yellow box in (g), where up to group 9 element 3 can be observed. Quantitative phase profiles along line *l*_1_ for group 8 and along line *l*_2_ for group 9 are plotted in Figure 3i1,i2, respectively. As presented in Figure 3i2, the group 9 element 3 can be distinguished, denoting the resolution at 0.775 μm (645.1 lp mm^−1^), the difference between the peak and valley of the curve is about 1 rad.

The possibility of realizing non‐priori digital refocusing using FD‐PR is attributed to the combination of two aspects. For one thing, the loss function formulated in the feature domain is more robust to noise signals, which makes our method take full advantage of valid information in the images. For another, the utilization of optimizers helps to escape from the local minima so that the optimal parameters can be found. With the enhanced reconstruction robustness of FD‐PR, blind recovery of large optical aberrations for FPM without prior knowledge of the aberrations becomes simple, and large aberrations up to 6π can be recovered by FD‐PR.

### 2.4 FD‐PR for Noise‐Free Coded Ptychography

In applications like ptychography and its variations, the propagator matrix **A** contains a key component denoting the wavefront of the probe beam that dominates the image's formation and reconstruction, but is hard to measure directly.^[^ ^50^ ^]^ Jointly optimizing for both propagator matrix **A** and desired wavefront **x** enables phase retrieval to convert the hardware challenges into algorithmic problems. FD‐PR can recover the system parameters given by the propagator matrix, **A**, in Equation (1) as well, potentially decreasing the requirements for optical system calibration.

**Figure 4:**
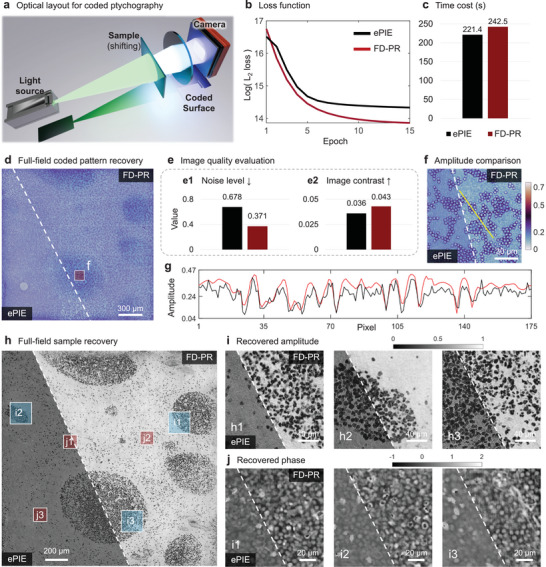
Phase retrieval for coded ptychography using FD‐PR. a) Sketch of the optical system. b) The evolution of loss for likelihood term. c) The execution time cost for ePIE and FD‐PR. d) The reconstructed coded pattern. e) Image quality measurement. e1) Image noise level. e2) Image contrast. f) Zoomed‐in image for the area in the red box in (d). g) Amplitude profiles along the yellow line in (f). h) Montage of full‐field recovery of the cell cluster using FD‐PR and ePIE. i) Recovered amplitude for ePIE and FD‐PR methods for areas marked by blue boxes (i1, i2, and i3) in (h). j) Recovered phase for ePIE and FD‐PR methods for areas marked by red boxes (j1, j2, and j3) in (h).

We conducted experiments on coded ptychography based on a public dataset^[^ ^51^ ^]^ as shown in **Figure** **4**a. Please refer Note S4 (Supporting Information) for experimental parameters. The setup enables lensless high‐throughput bio‐imaging where a coded pattern is placed between the sample and the detector. The coded pattern and the spatially shifted sample together encode the wavefront of the sample's exiting wave. Through the extended ptychographic iterative engine (ePIE), the wavefront for both the sample and the coded pattern can be reconstructed. The ePIE directly minimizes the pixel‐wise *L*_2_ loss function between the model‐predicted image and the collected image. In contrast, FD‐PR minimizes the *L*_1_ loss function based on the image's edge feature. Nevertheless, we use the *L*_2_ loss to evaluate the convergence of ePIE and FD‐PR algorithms. According to Figure 4b, FD‐PR converges faster than the ePIE, and obtains less *L*_2_‐loss value denoting better local minima. The time cost of FD‐PR is 242.5 s, longer than that of the ePIE with 221.4 s due to the implementation of Hessian regularization on both the recovered sample and the coded pattern.

The recovered coded pattern for the two methods is shown in Figure 4d. Image quality evaluation is quantified through Figure 4e1 noise level and Figure 4e2 image contrast. FD‐PR achieves the reduced noise level reconstruction to 0.371 (compared to 0.678 for ePIE) and improves the image contrast (0.043 vs 0.036 for ePIE). Zoomed‐in image for the area in the red box in Figure 4d is shown in Figure 4f where the contour of red blood cells for FD‐PR can be distinguished. The coded pattern recovered by ePIE is corrupted by noise signals, especially for the background area, as plotted in Figure 4g.

Full‐field sample recovery displays clear improvements by FD‐PR across the entire sample as illustrated in Figure 4h. The difference between the results of ePIE and FD‐PR are further highlighted for visual comparison, detailed in Figure 4i for amplitude, and j for phase. Recovered amplitude maps for the selected regions reveal that FD‐PR produces more uniform and noise‐free results compared to ePIE. Similarly, recovered phase maps show that FD‐PR achieves higher accuracy with reduced phase noise and white‐fog artifacts. Overall, the results demonstrate that FD‐PR outperforms ePIE in terms of noise suppression, image contrast enhancement, and reconstruction quality, making it a more reliable method for coded ptychography.

### 2.5 FD‐PR with Arbitrary Denoising Constraints

**Figure 5:**
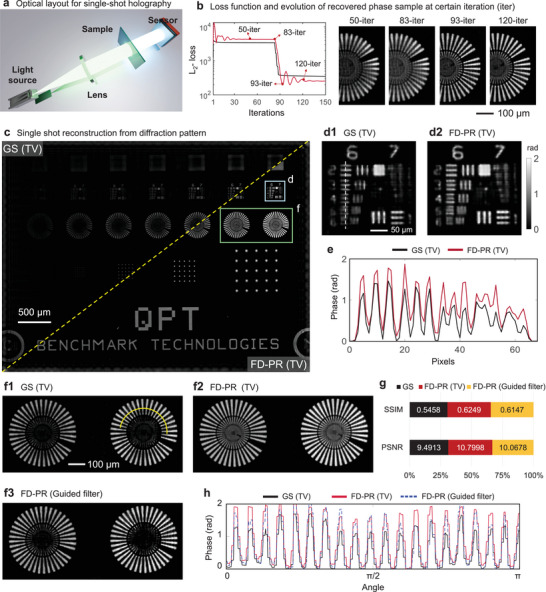
Single‐shot phase retrieval from in‐line holography. a) sketch of the optical set. b) Evolve of loss function for GS and FD‐PR methods, and the intermediate results of FD‐PR in certain iterations. c) Montage of results from GS algorithm and FD‐PR. d1,d2) Zoomed‐in pictures for the area in the blue box in (c). e) The quantitative phase profile for (d1) and (d2) along the white line in (d1). f1,f2) Zoomed‐in pictures for the area in the green box in (c), with additional results constrained by the guided filter. g) Comparison of the SSIM and PSNR of the three results. h) The quantitative phase profile along the yellow curve in (f1).

We conduct experiments using single‐shot phase retrieval for in‐line holography to show the flexibility of FD‐PR in combination with different types of constraints. Here, we merge the physical constraints and the image processing constraints such as denoising into FD‐PR to achieve single‐shot phase retrieval from the diffraction pattern of inline holography. The experimental layout is shown in **Figure** **5**a.

The experimental results are shown in Figure 5 which is compared with the conventional Gerchberg–Saxton (GS) algorithm with TV denoiser. As given in Figure 5b,c, both the GS algorithm and FD‐PR achieve single‐shot phase retrieval from the latent complex amplitude of the sample. The GS converges faster than FD‐PR, but the GS has a higher final loss than FD‐PR denoting that the GS algorithm falls into the local minimum. Under the acceleration of the YOGI optimizer,^[^ ^52^ ^]^ FD‐PR shows vibration properties before its convergence, in which the optimizer tries to get rid of local minima. Comparing Figure 5d1,d2, FD‐PR shows better visual reconstruction quality than that of the GS algorithm. The recovered resolution in the phase pattern of FD‐PR is finer than the GS as plotted in Figure 5e.

We tried the guided filter as the denoiser to erase the twin image in in‐line holography, as shown in Figure 5f3, which turns out that the guided filter can erase the twin image to recover the wheel‐like, pure‐phase structures. The guided filter can remove the diffuse conjugated signal and overcome the inherent physical symmetry of holographic reconstruction. The reconstruction quality using the guided filter is comparable to that of using total variations for the recovery of large structures as plotted Figure 5h. The evaluation based on PSNR and SSIM proves the reconstruction quality of the three methods, as given in Figure 5g. But the tiny structures are removed by the guided filter as shown in Figure 5f3. Fine‐tuning the parameter of smooth strength of the guided filter can save more tiny structures. In general, we proposed the eHIO for FD‐PR to provide the interface for more than one arbitrary constraint. The experiments here only show two constraints including the physical constraint applied to the amplitude of the results, and the image‐processing constraints including denoising and image smoothing to remove the twin‐image. More constraints can be applied according to the recovery requirements. Experimental parameters can be found in Note S4 (Supporting Information).

## 3 Discussion

Diverse phase retrieval algorithms share a common goal of seeking a solution through a non‐convex regression. The estimation of the desired wavefront is obtained from a series of intensity observations by maximizing both the likelihood function and the prior function. The likelihood function quantifies the error between the observation and the prediction generated from the forward model,^[^ ^53^ ^]^ which is typically defined by numerical wave diffraction, propagation, and intensity‐only detection. On the other hand, the prior function offers refinements to the resultant parameters. The refinements may involve applying empirical constraints or post‐processing techniques such as denoising or thresholding (see Note S5, Supporting Information, for detailed discussions). Therefore, phase retrieval necessitates three key components: the prior (constraint), the likelihood, and an effective solution to handle the optimization.

Most phase retrieval methods focus on implementations of different prior which introduces regularization or constraints on parameters. While a well‐selected prior can lead to elegant solutions, especially for ill‐posed cases, a better‐designed data likelihood term is more important than the prior,^[^ ^54^ ^]^ as likelihood provides the main driving force to the gradient descent (see Note S6, Supporting Information for detail discussions). This work focuses on the phase retrieval problem and reports a generalized phase retrieval engine (FD‐PR) based on feature‐domain consistency. FD‐PR is a non‐learning method with high interpretability compared to deep learning methods, every procedure and intermediate product of FD‐PR can be understood by researchers. FD‐PR can be regarded as an advanced non‐convex regularization which is composed of optical wave propagation processes, that may or may not be known. From the optimization point of view, non‐convex phase retrieval bears a resemblance to training a deep neural network in a supervised manner,^[^ ^55^ ^]^ implying that phase retrieval is similar to learning progress. Complex gradient descent can be an appropriate candidate and a generalizable approach for universal non‐convex optimization for phase retrieval. Fully developed optimization/learning strategies in deep learning, such as different optimizers to accelerate gradient descent and deep denoising prior, can be further adapted for phase retrieval (see Note S7, Supporting Information).

The proposed eHIO in FD‐PR extends the conventional HIO algorithm to more generalized conditions, demonstrating great stability, particularly with varying learning rates. An essential advantage of the HIO is its flexibility in providing more than one interface to accommodate arbitrary constraints in the phase retrieval process. FD‐PR establishes a generalized framework for feature‐domain likelihood. The **Θ** is the feature extractor that extracts the features from the scaled intensity data. FD‐PR was tested across several phase retrieval tasks, including Fourier ptychography, coded ptychography, and inline holography. The results show that FD‐PR can effectively extract and capture image features from raw data, making it less sensitive to outliers (such as vignetting effects) that are difficult to correct with denoising methods. Both visual and quantitative assessments of image quality show that FD‐PR produces better target wavefront in FPM and coded ptychography reconstruction based on feature likelihood, compared to widely adopted algorithms in each specific phase retrieval task. On average, the FD‐PR improves the resolution by a factor of 1.5 and reduces noise levels by a factor of 2 in four different phase retrieval tasks presented in Section 2, denoting its promising generalizability in a broad class of phase retrieval tasks. Moreover, FD‐PR is suitable for large‐scale phase retrieval as no inverse matrices calculation is required. The propagator matrix **A** and its complex complex conjugate can be calculated in advance if the optical layout is determined. Additionally, FD‐PR can be extended to solve wavefront shaping tasks such as beam designing,^[^ ^21^ ^]^ PSF engineering,^[^ ^56^ ^]^ and diffraction neural network optimization.^[^ ^57^ ^]^

Like other iterative‐based phase retrieval methods such as alternative projection,^[^ ^7^, ^26^, ^31^ ^]^ and proximal gradient,^[^ ^32^ ^]^ FD‐PR solves the phase through iterative optimization, which can limit its application in real‐time computational microscopy. Using CUDA programming optimized for a specific type of phase retrieval can accelerate the iterative optimization. Another limitation is the selection of feature extractors. This study demonstrates that using image features for phase retrieval can improve reconstruction quality. However, different feature extractors naturally identify different image features. The specific image features that most effectively contribute to phase retrieval can be investigated in future research. In the research presented here, we found that the edge feature leads to good‐quality phase retrieval since the image's edge is a universal low‐level image feature. Moreover, the wavelet transform is applicable as the wavelet transformation extracts different scales of the image's features as presented in Section 2.2, forming the feature pyramid.^[^ ^58^ ^]^ With the advance of deep learning, promising candidates of feature extractors can be trained from a large amount of image data.

In conclusion, FD‐PR is a general framework for phase retrieval tasks, which enables to exploration of potential likelihood functions, feature extractors, and constraints for diverse phase retrieval problems including but not limited to computational imaging, wavefront shaping, and beam designing. On the other hand, by assigning specific optical propagation operators such as positional shifting, or illumination patterns to every component of propagator **A**, one can design and modify the forward model of FD‐PR, which may introduce potential new optical layouts and applications. As the pseudo‐closed form gradient is provided, FD‐PR can be achieved by the automatic differentiation^[^ ^59^ ^]^ with differential programming that largely decreases the computational complexity of intricate systems such as diffraction neural networks. This may open new insights for wavefront shaping with further boosted quality and applications.

## 4 Experimental Section

### Experimental Parameters

All experimental parameters such as the illumination wavelength, and pixel size of the camera are available in Note S4. The loss function and the closed gradient for updating the parameters were deduced in detail. Codes related to this research are published online.

### Computational Platform

All the calculations were performed on a personal desktop with an Intel(R) Core(TM) i9‐12900K CPU @ 3.20GHz, 32GB of RAM, and Nvidia RTX 3090 graphic card, running the Windows 10Pro for Workstations. The code was written, compiled, and run in the MATLAB R2023b software. All the comparison studies on efficiency were performed in the same computational environment.

### Statistical Analysis

The data were presented as mean ± standard deviation. The software programs of MATLAB R2023b, ImageJ, and Microsoft Excel were utilized to perform the statistical analysis and create the graphs.

## Supporting Information

Supporting Information is available from the Wiley Online Library or from the author. The data supporting the results of this study will be available from the authors upon reasonable request. See Supplement Material for supporting content. Codes are available on Github.^[^ ^60^ ^]^

## Conflict of Interest

The authors declare no conflict of interest.

## Supporting information

Supporting Information

## Data Availability

The data that support the findings of this study are available from the corresponding author upon reasonable request.
