# Supplementary material for: High‐Fidelity Computational Microscopy via Feature‐Domain Phase Retrieval
Source: Adv Sci (Weinh). 2025 Feb 22;12(21):2413975. doi: 10.1002/advs.202413975 (PMC12140312; doi:10.1002/advs.202413975)
Supplement: Supplementary file 1 — Supporting Information [file ADVS-12-2413975-s001.pdf]

ADVANCED SCIENCE
Open Access

Supporting Information

for *Adv. Sci.*, DOI 10.1002/advs.202413975

High-Fidelity Computational Microscopy via Feature-Domain Phase Retrieval

*Shuhe Zhang, An Pan, Hongbo Sun, Yidong Tan and Liangcai Cao**

Supporting Information for

High-fidelity computational microscopy via feature-domain phase retrieval

Shuhe Zhang[1], An Pan[2,3], Hongbo Sun[1], Yidong Tan[1], and Liangcai Cao[1]

[1] Department of Precision Instruments, Tsinghua University, Beijing 100084, China

[2] State Key Laboratory of Transient Optics and Photonics, Xi'an Institute of Optics and Precision Mechanics, Chinese Academy of Sciences, Xi'an 710119, China

[3] University of Chinese Academy of Sciences, Beijing 100049, China

Table of Contents

Supporting Information for *High-fidelity computational microscopy via feature-domain phase retrieval* 1
Note S1 Feature-domain phase retrieval 3
Note S2 Extended hybrid input-output 5
Note S3 Optimization with complex back-propagation 7
Note S4 Experimental setup 9
Note S5 Constraint block 13
Note S6 Likelihood block 14
Note S7 Optimizers for complex variables 16
Note S8 On the scaling function $\mathcal{S}$ 19
Note S9 Additional experimental results for FD-PR on FPM 21

Note S1 Feature-domain phase retrieval

We treat the feature-domain phase retrieval (FD-PR) as a neural network but governed by linear optical processes, that is, the propagation of optical waves and how the final intensity measurements are formed. The computational graph is determined based on the principles of wave propagation in ideal conditions:

$$\mathbf{I} = |\mathbf{A}\mathbf{x}|^2 , \tag{S1}$$

in which the propagator $\mathbf{A}$ may be separated into several linear operators. For example $\mathbf{A} = \mathbf{FPM}$ in ptychography, where $\mathbf{F}$, $\mathbf{P}$ and $\mathbf{M}$ denote Fourier transform, illuminating by probe beam, and selection of certain image area, respectively.

Maximum A Posteriori

The wavefront recovery from intensity observation is a Maximum A Posteriori (MAP) problem where the posteriori $\mathcal{P}\left(\mathbf{x}, \mathbf{A}|\mathbf{I}_1^{obs}, \mathbf{I}_2^{obs}, \mathbf{I}_3^{obs}, \cdots, \mathbf{I}_N^{obs}\right)$ is required to be maximized. Following Bayesian and assuming that there is no relationship among $\mathbf{I}_n^{obs}$, we have

$$\text{argmax}\mathcal{P}\left(\mathbf{x}, \mathbf{A}|\mathbf{I}_1^{obs}, \mathbf{I}_2^{obs}, \mathbf{I}_3^{obs}, \cdots, \mathbf{I}_N^{obs}\right) = \text{argmax}\frac{\prod_{n=1}^{N} \mathcal{P}\left(\mathbf{I}_n^{obs}|\mathbf{x}, \mathbf{A}\right)\mathcal{P}\left(\mathbf{x}\right)\mathcal{P}\left(\mathbf{A}\right)}{\prod_{n=1}^{N} \mathcal{P}\left(\mathbf{I}_n^{obs}\right)}, \tag{S2}$$

where $\mathcal{P}\left(\mathbf{I}_n^{obs}|\mathbf{x}, \mathbf{A}\right)$ is the likelihood distribution. $\mathcal{P}(\mathbf{x})$ and $\mathcal{P}(\mathbf{A})$ are priori distribution for $\mathbf{x}$ and $\mathbf{A}$.

Taking minus-logarithm to both sides of Eq. (S2) and omitting terms that are not related to the parameters $\mathbf{x}$ and $\mathbf{A}$, we have the log-Posteriori given as

$$\begin{aligned}\text{argmax}\mathcal{P}\left(\mathbf{x}, \mathbf{A}|\mathbf{I}_1^{obs}, \mathbf{I}_2^{obs}, \mathbf{I}_3^{obs}, \cdots, \mathbf{I}_N^{obs}\right) &\propto \text{argmin} - \log\left[\mathcal{P}\left(\mathbf{x}, \mathbf{A}|\mathbf{I}_1^{obs}, \mathbf{I}_2^{obs}, \mathbf{I}_3^{obs}, \cdots, \mathbf{I}_N^{obs}\right)\right] \\ &\propto \text{argmin} - \sum_{n=1}^{N} \log\mathcal{P}\left(\mathbf{I}_n^{obs}|\mathbf{x}, \mathbf{A}\right) - \log\mathcal{P}(\mathbf{x}) - \log\mathcal{P}(\mathbf{A}) .\end{aligned} \tag{S3}$$

The first term in Eq. (S3) is the log-likelihood function, which is also known as the fidelity term that provides the main gradient for FPM reconstruction. The second and third term is the log-priori function applied to parameters $\mathbf{x}$ and $\mathbf{A}$ respectively, which provides regularization on their solutions.

Generalized feature-domain likelihood

The geometrical meaning of the log-likelihood function is the "distance" between the prediction and observation. Leveraging the forward model in Eq. (S1), we propose the feature-domain forward model given as

$$\mathbf{\Theta}\mathcal{S}\left(\mathbf{I}_n^{pre}\right) = \mathbf{\Theta}\mathcal{S}\left(|\mathbf{A}_{n,m}\mathbf{x}|^2\right) . \tag{S4}$$

Given a function $\mathcal{D}$ that evaluates the distance between $\mathbf{I}_n^{pre}$ and $\mathbf{I}_n^{obs}$. The output of $\mathcal{D}$ is a scalar following exponential distribution: $\exp\left[-\mathcal{D}\left(\mathbf{I}_n^{pre}, \mathbf{I}_n^{obs}\right)\right]$. Substituting into Eq. (S3) yielding the loss function for feature-domain likelihood:

$$\mathcal{L}_{\text{Likelihood}}(\mathbf{x}) = \sum_{n=1}^{N} \mathcal{D}\left[\boldsymbol{\Theta}\mathcal{S}(\mathbf{I}_n^{obs}),\ \boldsymbol{\Theta}\mathcal{S}(|\mathbf{A}_{n,m}\mathbf{x}|^2)\right],\ \mathbb{C}^{K_{in}} \longrightarrow \mathbb{R}. \tag{S5}$$

It is worth noting that the operator $\boldsymbol{\Theta}$ and $\mathcal{S}$ can be together regarded as a data preprocessing treatment but is embedded into the wavefront retrieval engine. Tab. S1 lists some (often used) distance functions and their derivatives.

Table S1. Sample distance functions and their derivative.$\mathbf{x}$: modal prediction, and $\mathbf{y}$: observation.

$\mathcal{D}(\mathbf{x},\mathbf{y})$		Derivative w.r.t $\mathbf{x}$
$\|\mathbf{x}-\mathbf{y}\|_2^2$	L_2-loss (Gaussian distribution noise)	$2(\mathbf{x}-\mathbf{y})$
$\|\mathbf{x}-\mathbf{y}\|_1$	L_1-loss (Laplacian distribution, sparse)	$\text{sign}(\mathbf{x}-\mathbf{y})$
$\|\mathbf{x}-\mathbf{y}\|_p^p$	L_p-loss (Hyper-Laplacian)	$p\|\mathbf{x}-\mathbf{y}\|^{p-1}\text{sign}(\mathbf{x}-\mathbf{y})$
$\mathbf{x}-\mathbf{y}\log\mathbf{x}$	Poisson-loss (Poisson distribution)	$1-\frac{\mathbf{y}}{\mathbf{x}}$
**More**		

The forward model in Eq. (S1) gives the reciprocity to both $\mathbf{x}$ and $\mathbf{A}_{n,m}$ as their position can be exchanged. This allows us to correct the system parameters in $\mathbf{A}_{n,m}$ by calculating the derivative w.r.t $\mathbf{A}_{n,m}$ from Eq. (S5) during the wavefront reconstruction. To do this, we assumed that $\mathbf{A}_{n,m}$ is further decomposed as a series of linear operations such that

$$\mathbf{A}_{n,m} = \mathcal{A}_k\mathcal{A}_{K-1}\cdots\mathcal{A}_k\cdots\mathcal{A}_2\mathcal{A}_1 = \prod_{k=K}^{1}\mathcal{A}_k. \tag{S6}$$

With Eq. (S6), each sub-component of $\mathbf{A}_{n,m}$ is parameterized and can be optimized.

Note S2 Extended hybrid input-output

The prior distribution in Eq. (S3) of the $\mathbf{x}$, and $\mathbf{A}$ serve as a regularization and refinements mechanism for improving the solution. Such regularization or refinements can be regarded as an arbitrary constraint that is imposed on the wavefront. More generally, the constraint can either originate from the physical aspect, such as spatial constraint given by an aperture or originate from digital aspect digital aspects involving image processing techniques like denoising or thresholding. We found inspiration in Fienup's hybrid input-output algorithm (HIO) [1, 2] and extended it to the FD-PR, enabling the incorporation of arbitrary constraints to enhance the optimization.

Let $\mathcal{C}(\mathbf{x})$ be the modified $\mathbf{x}$ after a given constraint $\mathcal{C}$ was performed with in the area $\mathcal{M}$. According to the HIO, the desired change on output $\mathbf{x}$ is given as

$$\delta\mathbf{x}(x) = \begin{cases} 0, & x \notin \mathcal{M} \\ \mathbf{x} - \mathcal{C}(\mathbf{x}), & x \in \mathcal{M}. \end{cases}$$

so that $\mathbf{x} - \delta\mathbf{x}(x) = \mathcal{C}(\mathbf{x})$. Based on HIO algorithm, $\mathcal{C}(\mathbf{x})$ is a new variable that is unrelated to $\mathbf{x}$, and the next input of $\mathbf{x}$ for optimization is modified by

$$\mathbf{x}^{t+1} = \mathbf{x}^t - \beta\delta\mathbf{x}(x) = \begin{cases} \mathbf{x}^t, & x \notin \mathcal{M} \\ \mathbf{x}^t - \beta\left[\mathbf{x} - \mathcal{C}(\mathbf{x})\right], & x \in \mathcal{M} \end{cases}.$$

This equation embodies two insights: from the physical aspect, it compensates for constraint violations by adjusting the input with strength β, which is the core idea of Fienup's HIO algorithm. From the mathematical aspect, it represents a gradient descent process to minimize energy between $\mathbf{x}^{t+1}$ and $\mathbf{x}^t$, guided by the associated loss function: $\mathcal{L}(\mathbf{x}^t) = \left\|\left|\mathbf{x}^{t+1} - \mathbf{x}^t\right|\right\|_2^2 = \||\mathbf{x} - \mathcal{C}(\mathbf{x})|\|_2^2, \quad \mathbb{C}^{K_{in}} \longrightarrow \mathbb{R}$. Moreover, one can not only minimize the absolute value of the difference but can also minimize the gamma-corrected difference:

$$\mathcal{L}(\mathbf{x}^t; p) = \||\mathbf{x} - \mathcal{C}(\mathbf{x})|^p\|_2^2. \quad p \in \mathbb{R}^+. \tag{S7}$$

The gamma correction is able to re-scale the strength of constraint point-wisely according to the energy. In additional, the constraint operation can be applied to parameters $\mathcal{A}_k$.

After incorporating Eq. (S7) into the FD-PR framework, the loss function of FD-PR is written as

$$\begin{aligned} \mathcal{L}_{\text{FD-PR}}(\mathbf{x}, \mathcal{A}_k) = \sum_{n=1}^{N} \mathcal{D}\left[\mathbf{\Theta}\mathcal{S}(\mathbf{I}_n^{obs}),\ \mathbf{\Theta}\mathcal{S}(|\mathbf{A}_{n,m}\mathbf{x}|^2)\right] \qquad (\mathcal{L}_{\text{Likelihood}}) \\ + \alpha \||\mathbf{x} - \mathcal{C}(\mathbf{x})|^p\|_2^2 + \sum_{k=1}^{K} \beta_k\ \||\mathcal{A}_k - \mathcal{C}(\mathcal{A}_k)|^p\|_2^2 \qquad (\mathcal{L}_{\text{Constrain}}), \end{aligned} \tag{S8}$$

all parameters can be learned from minimizing the FD-PR loss function, Eq. (S8), through complex back-propagation.

The FD-PR framework incorporates the proposed extended HIO, serving as a generalized Gerchberg-Saxton (GS) algorithm (Fig. S1). The GS alternates between object and Fourier domain constraints to

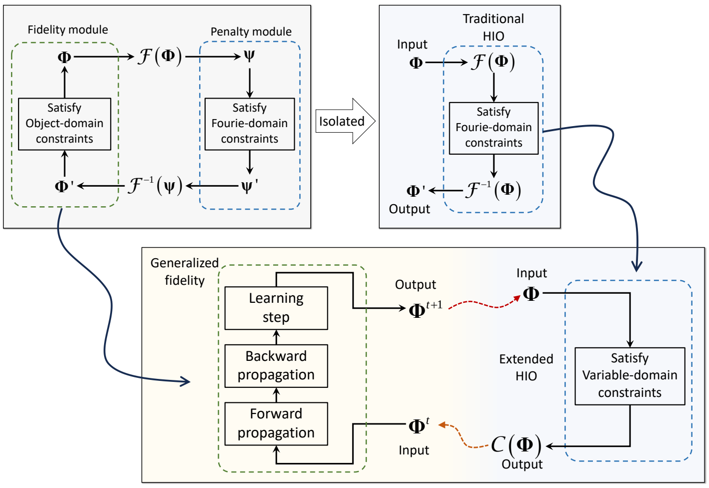

Fig. S1. Relationship between GS-algorithm, HIO-algorithm, extended HIO and how it is incorporated in FD-PR.

minimize the error between prediction and observation. If the object-domain constraints was treated as the likelihood-optimization, it would be natural to treat the Fourier-domain constraints as the prior/penalty-optimization. Further, the HIO isolates the penalty-optimization, and introduces customized constraints based on physical conditions, such as area shape support or intensity dynamic range thresholding.

In FD-PR, we optimize the likelihood using complex back-propagation, treating $\mathbf{x}^t$ as the input and $\mathbf{x}^{t+1}$ as the output after each gradient step. By reintroducing HIO, we insert custom constraints on $\mathbf{x}$ during gradient descent, leading to a refined penalty function that enhances the reconstruction quality.

Overall, the extended HIO in FD-PR combines GS-like behavior with back-propagation optimization, allowing for arbitrary constraints on the latent complex amplitude $\mathbf{x}$. This integration provides generalization and experimental robustness for wavefront retrieval.

Note S3 Optimization with complex back-propagation

Due to the non-convexity and non-linear property of the optimization, parameters $\mathcal{A}_k$ and $\mathbf{x}$ are learned from $\mathbf{I}_n^{obs}$ by minimizing $\mathcal{L}_{\text{FD-PR}}$ through complex gradient descent method. $\mathcal{C}(\mathbf{x})$ is regarded as a constant when calculates the gradient. As all operations in Eqs. (S5) and (S7) are assumed to be differentiable, the complex gradient of Eq. (S5) can be calculated using the $\mathbb{CR}$-calculus [3].

Learning for parameters. The derivative w.r.t. $\mathbf{x}$ and $\mathcal{A}_k$ are calculated as

$$
\begin{aligned}
\nabla_{\mathbf{x}}\mathcal{L}_{\text{FD-PR}} &= \alpha\frac{\mathbf{x}-\mathcal{C}(\mathbf{x})}{|\mathbf{x}-\mathcal{C}(\mathbf{x})|^{2-2p}+\epsilon} + \sum_{n=1}^{N}\sum_{m=1}^{M}\mathbf{A}_{n,m}^{\dagger}\mathbf{W}_{n,m}, \\
\nabla_{\mathcal{A}_k}\mathcal{L}_{\text{FD-PR}} &= \beta_k\frac{\mathcal{A}_k-\mathcal{C}(\mathcal{A}_k)}{|\mathcal{A}_k-\mathcal{C}(\mathcal{A}_k)|^{2-2p}+\epsilon} + \left(\prod_{l=k-1}^{1}\mathcal{A}_l\mathbf{x}\right)^{\dagger}\prod_{l=k+1}^{K}\mathcal{A}_l^{\dagger}\mathbf{W}_{n,m}.
\end{aligned}
\tag{S9}
$$

where ϵ is a small value in the case of divided by zeros, and

$$
\mathbf{W}_{n,m} = \text{Diag}\left[(\mathbf{A}_{n,m}\mathbf{x})\circ\mathcal{S}'\left(\mathbf{I}_n^{cal}\right)\right]\boldsymbol{\Theta}^{\dagger}\mathcal{D}'\left[\boldsymbol{\Theta}\mathcal{S}\left(\mathbf{I}_n^{cal}\right),\ \boldsymbol{\Theta}\mathcal{S}\left(\mathbf{I}_n^{obs}\right)\right]. \tag{S10}
$$

here $\dagger$ denotes the conjugate transpose (Hermitian transpose).

Eq. (S10) hides the concept of difference map (DM) in the derivative of $\mathcal{D}$. It tells how the changes of distance between $\mathbf{I}_n^{obs}$ and $\mathbf{I}_n^{cal}$ are back-propagated and are finally contributed to a change in $\mathbf{x}$. The $\boldsymbol{\Theta}$ denotes extracting the feature from the scaled-intensity data while $\boldsymbol{\Theta}^{\dagger}$ denotes its transpose operation. In addition, the physical meaning of $\mathbf{A}_{n,m}^{\dagger}$ in Eq. (S9) is inverting all-optical processes when the image was formed. In the optical aspect, this is literally 'back-propagating' the wavefront to the source plane $\mathbf{x}$. For example, the Fraunhofer propagator that diffracts the input wave to the output wave is described as the Fourier transform, while its Hermitian transpose denotes the inverse Fourier transform which exactly diffracts the output wave back to the input wave.

It is worth noting that Eq. (S9) calculates the batch gradient where all N training data (observed intensity) is taken into consideration for a single step. While one can also calculate the gradient from parts of the observation yielding the stochastic gradient in case of a large volume of training data. Similar to training a neural network, the learning process for FD-PR can be accelerated depending on the optimizers, namely the SGD, Adam [4], the AdaBelief [5], or the Lion [6] for updating of complex variables. After initialization of the parameters and image formation model, the parameters are updated iteratively. When the loss is smaller than a given threshold, the iteration stops with convergence.

In general, the FD-PR is organized into three main components, similar to a conventional NN:

1. FD-PR.forward(): in this component the engine forms the computational graph, and calculates the difference between model prediction and measurement according to the given image formation model and loss function.
2. FD-PR.backward(): in this component the gradients with respect to the parameters to be optimized are traced and computed.
3. FD-PR.step(): finally, the parameters are updated using complex variable optimizers.

Table S2. Wavefront retrieval engine

Input: Image formation model; parameter to be optimized ($\mathbf{x}$, $\mathcal{A}_k$); intensity observations $\mathbf{I}_n$; optimizer's parameters $(\gamma_1, \gamma_2, \eta)$; Cost function $\mathcal{D}$; Constraints $\mathcal{C}$ and constraints parameters

While $t \leq iter_{max}$

FD-PR.forward()

Calculating the loss function using Eq. (S8)

FD-PR.backward()

$\mathbf{x}_{\mathcal{C}} = \mathcal{C}(\mathbf{x}^t)$

$\mathcal{A}_{k,\mathcal{C}} = \mathcal{C}(\mathcal{A}_k^t)$

Calculating the gradient $\nabla_{\mathbf{x}}\mathcal{L}_{\text{FD-PR}}$ and $\nabla_{\mathcal{A}_k}\mathcal{L}_{\text{FD-PR}}$ using Eq. (S9) to Eq. (S10)

FD-PR.step()

$\mathbf{x}^{t+1} = \text{Optimizer}\left(\mathbf{x}^t, \nabla_{\mathbf{x}}\mathcal{L}_{\text{FD-PR}}\right)$

$\mathcal{A}_k^{t+1} = \text{Optimizer}\left(\mathcal{A}_k^t, \nabla_{\mathcal{A}_k}\mathcal{L}_{\text{FD-PR}}\right)$

$t = t + 1$

End While

Output: refined complex amplitude $\mathbf{x}_{\mathcal{C}}$ and $\mathcal{A}_{k,\mathcal{C}}$

Note S4 Experimental setup

This section provides basic formulas and experimental parameters for content in the result section of the main text.

Fourier ptychography: The forward model for Fourier ptychography reads

$$\mathbf{I}_n^{pre} = \left|\mathbf{F}^{-1}\mathbf{P}\mathbf{M}_n\mathbf{F}\mathbf{x}\right|^2, \tag{S11}$$

where $\mathbf{x}$ is the complex amplitude of sample, $\mathbf{F}$ is the Fourier transform. $\mathbf{M}_n$ is the selection matrix for the corresponding LED illumination angle. $\mathbf{P}$ is the pupil function. The feature-domain likelihood loss function is given as

$$\mathcal{L}_{\text{Likelihood}} = \left\|\mathbf{\Theta}\sqrt{\mathbf{I}^{pre}} - \mathbf{\Theta}\sqrt{\mathbf{I}^{obs}}\right\|_1. \tag{S12}$$

The camera pixel size is 6.5 µm, and the NA = 0.1 (4×) for the objective lens. A total of 361 LEDs are used, placed 65 mm away from the sample, with a distance of 4 mm between two adjacent LED dots. The illumination wavelengths are 631 nm, 532 nm, and 488 nm for red, green, and blue LEDs. The USAF is illuminated only with red LEDs. Potential LED position misalignment exists and the raw image has severe vignetting and Gaussian noise.

According to the Eq. (2) in the main text, the derivative of the loss function w.r.t. parameters $\mathbf{x}$ is given as

$$\nabla_{\mathbf{x}}\mathcal{L} = \mathbf{F}^{-1}\mathbf{M}_n^{\top}\mathbf{P}^{\dagger}\mathbf{F}\left\{\text{Diag}\left[\mathbf{\Theta}^{\top}\text{sign}\left(\mathbf{\Theta}\sqrt{\mathbf{I}^{pre}} - \mathbf{\Theta}\sqrt{\mathbf{I}^{obs}}\right)\right]\frac{\mathbf{F}^{-1}\mathbf{P}\mathbf{M}_n\mathbf{F}\mathbf{x}}{|\mathbf{F}^{-1}\mathbf{P}\mathbf{M}_n\mathbf{F}\mathbf{x}|}\right\}, \tag{S13}$$

and the derivative w.r.t. parameters $\mathbf{P}$ is given as

$$\nabla_{\mathbf{P}}\mathcal{L} = \left(\mathbf{M}_n\mathbf{F}\mathbf{x}\right)^{\dagger}\mathbf{F}\left\{\text{Diag}\left[\mathbf{\Theta}^{\top}\text{sign}\left(\mathbf{\Theta}\sqrt{\mathbf{I}^{pre}} - \mathbf{\Theta}\sqrt{\mathbf{I}^{obs}}\right)\right]\frac{\mathbf{F}^{-1}\mathbf{P}\mathbf{M}_n\mathbf{F}\mathbf{x}}{|\mathbf{F}^{-1}\mathbf{P}\mathbf{M}_n\mathbf{F}\mathbf{x}|}\right\}. \tag{S14}$$

Eqs. (S13) and (S14) are used to update the parameters based on gradient descent.

Coded ptychography: The forward model for coded ptychography is written as [7]

$$\mathbf{I}_n^{pre} = \mathbf{D}\left|\mathbf{H}_2\mathbf{T}\mathbf{M}_n\mathbf{H}_1\mathbf{x}\right|^2. \tag{S15}$$

$\mathbf{x}$ is the sample wavefront. $\mathbf{H}_1$ and $\mathbf{H}_2$ are two propagators (angular spectrum diffraction) that diffract the input wavefront to a certain distance. $\mathbf{M}_n$ is the n-th shift operator, and $\mathbf{T}$ is the coded pattern. $\mathbf{D}$ is the down-sample operator denoting the discrete sampling of the detector. The goal is to solve both $\mathbf{x}$ and $\mathbf{T}$ from a series of observation of $\mathbf{I}_n^{obs}, n = 1, 2, 3, \cdots$.

We use the Gamma correction to be function $\mathcal{S}$ and use the image's gradient to form the feature-domain likelihood loss function where $\mathcal{L}_{\text{Likelihood}} = \|\nabla(\mathbf{I}_n^{pre})^{\gamma} - \nabla(\mathbf{I}_n^{obs})^{\gamma}\|_1$, γ is the gamma-correction parameter. The second-order TV regularization (Hessian regularization) [8] is applied to both sample $\mathbf{x}$ and the coded pattern $\mathbf{T}$. Other parameters can be found in the public dataset [7].

According to the Eq. (2) in the main text, the derivative of the loss function w.r.t. parameters $\mathbf{x}$ is given as

$$\nabla_{\mathbf{x}}\mathcal{L} = \mathbf{H}_1^{\dagger}\mathbf{M}_n^{\top}\mathbf{T}^{\dagger}\mathbf{H}_2^{\dagger}\text{Diag}\left\{\mathbf{D}^{\top}\frac{\nabla^{\top}\text{sign}\left[\nabla\left(\mathbf{I}_n^{pre}\right)^{\gamma} - \nabla\left(\mathbf{I}_n^{obs}\right)^{\gamma}\right]}{\left(\mathbf{D}\left|\mathbf{H}_2\mathbf{T}\mathbf{M}_n\mathbf{H}_1\mathbf{x}\right|^2\right)^{(1-\gamma)}}\right\}\left(\mathbf{H}_2\mathbf{T}\mathbf{M}_n\mathbf{H}_1\mathbf{x}\right), \tag{S16}$$

and the derivative w.r.t. parameters $\mathbf{P}$ is given as

$$\nabla_{\mathbf{T}}\mathcal{L} = (\mathbf{M}_n\mathbf{H_1}\mathbf{x})^{\dagger}\,\mathbf{H}_2^{\dagger}\text{Diag}\left\{\mathbf{D}^{\top}\frac{\nabla^{\top}\text{sign}\left[\nabla\left(\mathbf{I}_n^{pre}\right)^{\gamma}-\nabla\left(\mathbf{I}_n^{obs}\right)^{\gamma}\right]}{\left(\mathbf{D}\left|\mathbf{H}_2\mathbf{T}\mathbf{M}_n\mathbf{H}_1\mathbf{x}\right|^2\right)^{(1-\gamma)}}\right\}(\mathbf{H}_2\mathbf{T}\mathbf{M}_n\mathbf{H}_1\mathbf{x})\,. \tag{S17}$$

Eqs. (S16) and (S17) are used to update the parameters based on gradient descent for coded ptychography.

Hessian regularization: The Hessian regularization is an additional module that is solved independently of the gradient descent. For any given noise-corrupted image denoted by $\mathbf{s}$, the Hessian regularization aims to solve the following optimization problem:

$$\text{argmin}_{\mathbf{o}}\left\|\mathbf{s}-\mathbf{o}\right\|_2^2+\alpha\left(\left\|\nabla_{xx}\mathbf{o}\right\|_1+\left\|\nabla_{yy}\mathbf{o}\right\|_1+2\left\|\nabla_{xy}\mathbf{o}\right\|_1\right), \tag{S18}$$

where $\nabla_{xx} = [-1, 2, -1]$ denotes the convolution kernel of the second-order gradient of an image in x-direction. $\nabla_y = [-1, 2, -1]^{\top}$ denotes the convolution kernel of the second-order gradient of an image in y-direction. $\nabla_{xy} = [-1, 1; 1, -1]$ denotes the convolution kernel of the second-order gradient of an image in x and y direction.

Eq. (S18) is solved using the half-quadratic splitting method [9]. In introducing three auxiliary variables, $\mathbf{G}_{xx}$, $\mathbf{G}_{yy}$, $\mathbf{G}_{xy}$ w.r.t. $\nabla_{xx}\mathbf{o}$, $\nabla_{yy}\mathbf{o}$, $\nabla_{xy}\mathbf{o}$, the original optimization problem is split into four sub-optimization problems given as

$$\begin{cases}\text{argmin}_{\mathbf{o}}\left\|\mathbf{s}-\mathbf{o}\right\|_2^2+\alpha_0\left(\left\|\nabla_{xx}\mathbf{o}-\mathbf{G}_{xx}\right\|_2^2+\left\|\nabla_{yy}\mathbf{o}-\mathbf{G}_{yy}\right\|_2^2+2\left\|\nabla_{xy}\mathbf{o}-\mathbf{G}_{xy}\right\|_2^2\right)\\ \text{argmin}_{\mathbf{G}_{xx}}\left\|\nabla_{xx}\mathbf{o}-\mathbf{G}_{xx}\right\|_2^2+\dfrac{\alpha}{\alpha_0}\left\|\mathbf{G}_{xx}\right\|_1\\ \text{argmin}_{\mathbf{G}_{yy}}\left\|\nabla_{yy}\mathbf{o}-\mathbf{G}_{yy}\right\|_2^2+\dfrac{\alpha}{\alpha_0}\left\|\mathbf{G}_{yy}\right\|_1\\ \text{argmin}_{\mathbf{G}_{xy}}\left\|\nabla_{xy}\mathbf{o}-\mathbf{G}_{xy}\right\|_2^2+\dfrac{\alpha}{\alpha_0}\left\|\mathbf{G}_{xy}\right\|_1\end{cases}. \tag{S19}$$

The first subproblem in Eq. (S19) is pure quadratic and is solved direction by setting its derivative w.r.t. $\mathbf{x}$ to zero, and the solution is given as

$$\mathbf{o} = \frac{\mathbf{s}+\alpha_0\left(\nabla_{xx}^{\top}\mathbf{G}_{xx}+\nabla_{yy}^{\top}\mathbf{G}_{yy}+2\nabla_{xy}^{\top}\mathbf{G}_{xy}\right)}{1+\alpha_0\left(\nabla_{xx}^{\top}\nabla_{xx}+\nabla_{yy}^{\top}\nabla_{yy}+2\nabla_{xy}^{\top}\nabla_{xy}\right)}. \tag{S20}$$

The second to the fourth subproblems are solved using the soft-threshold method, and the solutions are given as

$$\begin{cases}\mathbf{G}_{xx} = \text{sign}\left(\nabla\mathbf{o}_{xx}\right)\max\left(\left|\nabla\mathbf{o}_{xx}\right|-\frac{\alpha}{\alpha_0},0\right)\\ \mathbf{G}_{xx} = \text{sign}\left(\nabla\mathbf{o}_{yy}\right)\max\left(\left|\nabla\mathbf{o}_{yy}\right|-\frac{\alpha}{\alpha_0},0\right)\\ \mathbf{G}_{xy} = \text{sign}\left(\nabla\mathbf{o}_{xy}\right)\max\left(\left|\nabla\mathbf{o}_{xy}\right|-\frac{\alpha}{\alpha_0},0\right)\end{cases}. \tag{S21}$$

Based on Eqs. (S20) and (S21), the flowchart for the Hessian regularization is The execution time of the Hessian regularization for different sizes of input images is tested. Each test is repeated 20 times and the average execution time and standard deviation are calculated. Tab. S3

Aberration correction: Experimental studies are conducted on blind aberration recovery for quantitative phase target (QPT). A circular LEDs panel consisting of a total of 93 LEDs is utilized. The

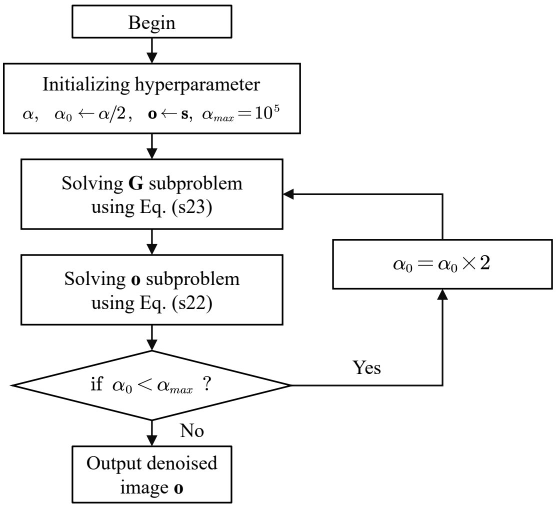

Fig. S2. Flowchart for Hessian regularization using the half-quadratic splitting method.

Table S3. Execution time of Hessian regularization for different input image size

	Image input size					
Size	256^2	512^2	1024^2	2048^2	4096^2	8192^2
Execution time (s)	0.0237 $\pm$ 0.0063	0.0323 $\pm$ 0.0220	0.0707 $\pm$ 0.0013	0.2964 $\pm$ 0.0609	1.1084 $\pm$ 0.0687	4.9437 $\pm$ 0.0690

panel has 6 LED rings, with differences in the radius of each ring set at 9 mm. The arrangement, from inner to outer rings, includes 1, 8, 12, 16, 24, and 32 LED dots, respectively. The panel is placed 110 mm away from the sample, achieving maximum illumination NA of $\text{NA}_{ill} = 0.316$. The synthetic NA reaches to 0.41. The central wavelength is 0.523 µm. An objective lens with a magnification of $\times 4$ and NA of 0.1 is employed.

For the simulation study, the coefficients of the Zernike functions are randomly assigned different values, resulting in 50 groups of aberrations ranging from 0 to 2π. Gaussian noise with a standard deviation of 0.0001 is added to simulate real-world noise. Each group underwent 10 rounds of testing, and the average PSNR and SSIM values were calculated for each group.

For experiments, the camera is MV-SUA133GM with 8-bit pixels and a pixel size of 4 µm. The structures within the QPT have a height of 150 nm and a refractive index of 1.56, resulting in a phase difference of approximately 0.99 rad.

Inline holography: The forward model for in-line holography reads

$$\mathbf{I}^{pre} = \mathbf{D} \left| \mathbf{H}_z \mathbf{x} \right|^2 . \tag{S22}$$

$\mathbf{D}$ is the down-sampling operator corresponding to the down-sampling of the camera sensor. $\mathbf{H}_z$ is the diffraction propagator that diffract the wave from z distance. The FD-PR loss function with multiple constraints is written as

$$\mathcal{L}_{\text{FD-PR}} = \|\sqrt{\mathbf{I}^{obs}} - \sqrt{\mathbf{D}|\mathbf{H}_z\mathbf{x}|^2}\|_2^2 + \alpha\||\mathbf{x} - \mathcal{C}_1(\mathbf{x})|\|_2^2 + \beta\||\mathbf{x} - \mathcal{C}_2(\mathbf{x})|\|_2^2. \tag{S23}$$

Here, $\mathcal{C}_1(\mathbf{x}) = \min(|\mathbf{x}|, I_0)\cdot\text{sign}(\mathbf{x})$, denoting the eHIO module providing physical constraint on amplitude of $\mathbf{x}$. I_0 is the amplitude limitation to force the uniformity of the pure-phase sample's spatial amplitude distribution. $\mathcal{C}_2(\mathbf{x})$ provides image-processing constraint which can be a denoiser applied to $\mathbf{x}$. Here the total variation (TV) denoiser is used to erase the twin-image caused by phase-conjugate wavefront [10]. The loss function $\mathcal{L}_{\text{FD-PR}}$ is a generic unconstrained type, which is directly optimized through complex gradient descent. The up-sampled rate is two and the optimizer is YOGI with default parameters. If the optimization is solved by ADMM, four more auxiliary variables corresponding to the first and the second constraint terms are required to solve the optimization problem, which is not efficient in computing and storage aspects.

The camera pixel size is 5.9 µm, illumination wavelength is 660 nm. The diffraction distance is 8.87 mm.

Reference-free Image noise and contrast evaluation: The noise level evaluation of a given image of $M \times N$ pixels, $\mathbf{x}$,is evaluated using reference-free global noise estimation [11] given as

$$\text{Noise level} = \frac{1}{MN}\sum_{n=1}^{n}\sum_{m=1}^{M}|\mathbf{x}\otimes\mathbf{k}| \tag{S24}$$

where $\mathbf{k} = [-1, 2, -1; 2, -4, 2; -1, 2, -1]$ is the Laplacian kernel. The evaluation takes advantage of the noise-sensitive property of the Laplacian kernel. A larger value denotes stronger noise levels.

For reference-free image contrast evaluation, we use the multi-scale contrast evaluation [12] given by

$$\text{Image contrast} = \frac{1}{8\#pixels\cdot\#levels}\sum_{\forall level}\left[\sum_{\forall pixels}\left(\sum_{8-neigh}|p_i - p_j|\right)\right] \tag{S25}$$

where $\#pixels$ denotes the total pixel numbers of the image, $\#levels$ denotes the total level of down-sampling, in each level the image is halved without pre-filtering. For a pixel centered at p_i, $8 - neigh$ denotes the surrounding 8 pixels in a 3×3 image patch. $|p_i - p_j|$ denotes the absolute difference between the center pixel p_i and a surrounding pixels p_j in the 3×3 image patch. In the experiments presented in the main text, we use 8 levels of down-sampling. The pixel numbers of retinal images in the databases are large enough for 8 levels of down-sampling.

Note S5 Constraint block

Most PR methods focus on implementations of different prior which introduces regularization or constraints on parameters. A well-selected prior can lead to an elegant solution, especially when the wavefront recovery optimization is ill-posed. The prior is typically chosen based on parameters' statistical properties or empirically chosen according to experiments. For instance, applying the Laplacian distribution assumption to the image's gradient results in the total variation regularization, which is widely used for tasks like image denoising, twin-image removal in digital holography, and spatiotemporal holography.

Beyond the total variation denoising, more generalized forms of prior, providing flexible interfaces for arbitrary regularization, attract research interest. Research on generalized prior has led to the development of image restoration frameworks such as plug-and-play alternative alternating direction method of multiplier (PnP-ADMM) and regularization by denoising (RED) [13]. However, most of these frameworks focus on real-valued signals. Only a few, like prDEEP [14] and large-scale phase retrieval (LPR), has been extended for complex-valued signals used in wavefront recovery. The prDEEP is based on RED, which has limited applications for complex signals, while LPR heavily relies on PnP-ADMM, which can only handle specific types of likelihood functions such as L_2-norm and L_1-norm. Additionally, wavefront recovery requires combining multiple types of constraints, such as physical constraints and denoising constraints, further complicating the use of ADMM due to the requirements of storage and frequent updating of multipliers.

We propose the eHIO which extends the conventional HIO algorithm into more generalized conditions as shown in Fig. S1. The eHIO differs from those methods in practical and mathematical aspects. Firstly, the decision to use the eHIO is based on its superior compatibility with the gradient descent routine. The eHIO demonstrates greater stability, particularly in scenarios involving varying learning rates. An essential advantage of the HIO is its flexibility in providing more than one interface for accommodating arbitrary constraints in the wavefront recovery process.

There is a fundamental difference between our eHIO and RED, from the mathematical aspect, in the way they try to minimize the constraint loss function. When $p = 1$ the mathematical formulation of our proposed eHIO constraint resembles the RED which was previously employed in the prDEEP framework [14]. The RED minimizes the loss $\mathbf{x}^\top [\mathbf{x} - \mathcal{C}(\mathbf{x})]$, yet, it results in a complex scalar which cannot be merged into the real-valued loss function when $\mathbf{x}$ is a complex-variable. In contrast, our eHIO minimizes the gamma-corrected optical energy associated with the difference between the input and the constrained output, represented as $\||\mathbf{x} - \mathcal{C}(\mathbf{x})|^p\|_2^2$. This is a real-valued multivariable complex function ensuring uniformity in the computation of gradients. Furthermore, the prDEEP uses the Fast Adaptive Shrinkage/Thresholding Algorithm (FASTA) to address the RED problem, which cannot recover complex-domain signals, leading to limited applications in practice. The FD-PR employs gradient descent and enables us to effectively handle non-quadratic and non-convex likelihood functions.

Note S6 Likelihood block

Despite the success of choosing prior, the likelihood plays a more pivotal role, dictating the success or failure of wavefront recovery [15]. From the geometric perspective, the likelihood measures the "distance" between the forward model prediction and experimental observation. This distance can have various geometrical interpretations. For instance, the Gaussian likelihood represents the Euclidean distance, while the Laplacian likelihood represents the Manhattan distance. From the Bayesian aspect, likelihood models the distribution of noise signal presence to the observed images [16]. The occurrence of outliers, mismatch of system parameters, and non-linearity of practical optical systems can all induce non-Gaussian or non-Poisson data.

Although a monochromatic image contains only two-dimensional data, the image's features such as corners, edges, and dots contain dramatically high-dimensional information that can be dug out through combinations of well-defined feature extractors. Compared to the image itself, the image's features are more robust to outliers degrading since the features are the inherent property. Establishing the feature-domain likelihood function using the image's edge feature can greatly improve the reconstruction quality for Fourier ptychography [17]. Studies in the field of computer vision [18, 19] also find the virtue of feature-domain optimization in bypassing image-domain challenging for real-valued signal.

The FD-PR establishes a generalized framework for feature-domain likelihood. The $\boldsymbol{\Theta}$ is the feature extractor that extracts the features from the scaled-intensity data while $\boldsymbol{\Theta}^{\dagger}$ denotes its transpose operation. This operator gives an interface for composing the loss function in the images' feature domain since not all pixels are useful and valid for wavefront recovery. $\boldsymbol{\Theta}$ can be manually designed or trained from a set of image data to extract certain features of the image, such as edge, textures, gradient, and resolution-in-variance bases [18]. Compared with the image itself, the image's features are more robust to problematic pixels such as noise, intensity fluctuation, and vignetting effect. For example, if the intensity fluctuation appears during the data collection, directly minimizing the loss function in the image domain is not efficient as the $\mathbf{x}$ will learn unexpected uneven intensity, possible yielding failure of reconstruction. Minimizing the loss function with the image's edge feature can bypass such intensity fluctuation since the edge feature is more invulnerable to intensity fluctuation.

Scaling function, $\mathcal{S}$, in Eq. (S4) provides non-linear mapping on the intensity measures such a Gamma correction, where $\mathcal{S}(\mathbf{x}) = \mathbf{x}^{\gamma}$. For single-intensity measurement, it is a good candidate for reconstruction with $\gamma = 0.5$. For wavefront recovery from multiple intensity measurements, the scaling function can impact the quality of wavefront recovery. Since not all images are at the same intensity level, the learning efficiency among one dataset is different. Images with brighter intensity normally provide a larger gradient than images with dimer intensity. The scale function can balance the intensity distribution within a single dataset, benefiting the learning process. For example, in Fourier ptychography where the dataset contains both bright-field and dark-field images, the amplitude-based likelihood works better than the intensity-based one in reconstructed resolution, as the dark-field images in the amplitude-based

case provide more gradient information than the intensity-based case. It is also shown that $\mathcal{S}(\mathbf{x}) = \log(\mathbf{x})$ may provide better reconstruction results compared to $\mathcal{S}(\mathbf{x}) = \mathbf{x}^{0.5}$. Please see **Supplement Note 8** for the discussion of scaling function.

The FD-PR enables customized data likelihood in the image's feature domain, which is not restricted to the moderate L_2-distance widely used in the LPR and other phase retrieval methods. The feature-extraction operator and scaling function provide the interface for flexible data augmentation and embedded pre-processing which allow the engine to make full use of the input data information. Such customized data likelihood allows measuring the data distance in different geometrical ways, and the engine can be optimized for certain wavefront recovery tasks based on data statistical features. Like training a neural network based on different loss functions [20], an appropriate data likelihood can accelerate the wavefront recovery, and gain better wavefront recovery and image restoration qualities. Moreover, the data likelihood function can also be learned and specialized for certain tasks using the AutoLoss. The FD-PR then records and calculates the complex gradient from the data likelihood for back-propagation. The gradient for all N training data (observed intensity) can be taken into consideration within a single step. One can calculate the gradient from parts of the observation yielding the stochastic batch gradient in case of a large volume of training data.

Note S7 Optimizers for complex variables

From the Gerchberg–Saxton (GS) algorithm, and hybrid input-output (HIO) algorithm to the recently developed Wirtinger-calculus, phase lift, LPR algorithms, and deep-learning methods, each optimization algorithm is only suitable for specific applications, and cannot be generalized. The PR algorithms varied in their definition, implementation, and underlying structures. Faced with the challenge of non-convexity in wavefront recovery, a traditional workaround in literature has been to modify the problem formulation itself, relaxing the non-convex to a convex problem, so that existing tools can be readily applied [21].

Such relaxing changes the problem drastically and omits the virtue of non-convex. The relaxed formulation has limited solutions, and cannot handle more intricate likelihood distribution such as Poisson likelihood. From the optimization point of view, non-convex wavefront recovery bears a resemblance to training a deep neural network in a supervised manner [22], implying that wavefront recovery is also learning progress. Complex gradient descent can be an appropriate candidate and a generalizable approach for universal non-convex optimization for wavefront recovery. Fruitful optimization/learning strategies, such as developed optimizers, in the field of deep learning can be further adapted for wavefront recovery.

Similar to training an NN, the learning process for FD-PR can also be accelerated depending on the optimizers, e.g. momentum. However, as stated in the PyTorch documentation that *all the existing optimizers work out of the box with complex parameters*, a little modification should be adapted to the optimizers since the loss function is complex-variable and non-convex.

In this section we modified three off-the-shelf optimizers, namely the Adam [4], the NAdam [23], the AdaBelief [5], and the Lion [6] for updating of complex variables.

Adam optimizers: For the Adam and the NAdam optimizer, we only have to modify the calculation of the second-moment term as listed in Tab. S4, where η is the learning rate, and ϵ is a small scalar to avoid the divided by zeros. As given in Tab. S4, the second-moment estimation uses the intensity of the complex gradient.

Difference from the optimizer for the real-valued parameter where the gradient-squared is directly computed, the squared absolute value should be calculated for the complex-valued parameter. The second moment estimation is adapted from the RMSProp optimizer where the geometrical length of the historical gradient vector is calculated to modify the learning rate. However, for a complex vector, the geometrical length should be calculated from its modules (absolute value, or intensity).

AdaBelief optimizer: Similar modification applied to the AdaBelief optimizer. The second term in Adabelief optimizer denotes the belief in gradient direction which uses the intensity of the difference between the first moment estimation and the complex gradient.

Lion optimizer: The Lion optimizer is similar to the SIGNSGD optimizer where the gradient vector is normalized in an-isotropic manner [24]. For complex variables, the 'sign' function remaps the complex variable onto a unit circle, with identical angles. Accordingly, the parameter $\mathbf{x}$ is updated using the

normalized amplitude gradient as marked in **Optimizer 3** in Tab. S4. In general, The algorithm for our proposed complex amplitude reconstruction algorithm is summarized in Tab. S2.

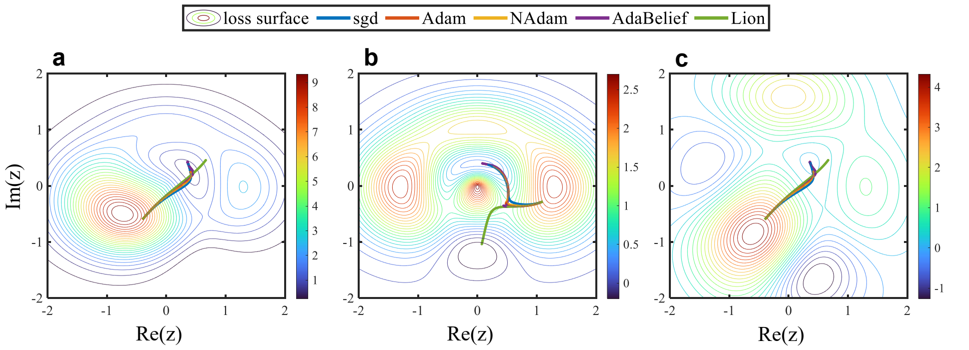

(a) $$\begin{aligned} f(z,\overline{z}) &= 8\left[1-\Re(z)\right]^2 \exp\left[-|z|^2-\Im^2(z)-2\Im(z)-1\right] \\ &- \frac{7}{5}\left[|z|-5|z|^3-5\Im^5(z)\right]\exp\left[-|z|^2-\Im^2(z)\right] \\ &- \frac{1}{3}\exp\left[-|z|^4-2|z|^2-\Im^2(z)-1\right] \end{aligned}$$

(b) $$\begin{aligned} f(z,\overline{z}) &= 8\left(1-|z|\right)^2 \exp\left[-|z|^2-\Im^2(z)-2\Im(z)-1\right] \\ &- \frac{7}{5}\left[|z|-5|z|^3-5\Im^5(z)\right]\exp\left[-|z|^2-\Im^2(z)\right] \\ &- \frac{1}{3}\exp\left[-|z|^4-2|z|^2-\Im^2(z)-1\right] \end{aligned}$$

(c) $$\begin{aligned} f(z,\overline{z}) &= 8\left[1-\Re(z)\right]^2 \exp\left[-|z|^2-\Im^2(z)-2\Im(z)-1\right] \\ &- \frac{7}{5}\left[|z|-5|z|^3-5\Im^5(z)\right]\exp\left[-|z|^2-\Im^2(z)\right] \\ &- \frac{1}{3}\exp\left[-|z|^4-2|z|^2-\Im^2(z)-1\right] \end{aligned}$$

Fig. S3. Optimization process for three loss surfaces.

The performance for these optimizers on (1D-case) different loss surfaces is plotted in Fig. S3. All loss surfaces are real-valued, complex functions for 1D complex input variable. The optimziers parameters are given as $\gamma_1 = 0.9$, $\gamma_2 = 0.99$, and $\eta = 0.01$. A total of 100 iterations are computed. Accordingly, the Adam/NAdam and AdaBelief converge to a similar local minimum but with different speeds. The AdaBelief is generally faster than Adam/NAdam, while the Lion updates the parameter even faster than Adam/NAdam and AdaBelief. However, the latter can overcome the local minimum, looking for a further potential local minimum. The choice of optimizers for wavefront retrieval depends on the task and certain applications. For accurate calculation, the Adam is preferred, while for fast convergence, the Lion is a good candidate.

Table S4. Optimizers for complex variable

Optimizer 1: Complex Adam (NAdam)

Initialize $\mathbf{x}^0$, $\mathbf{u}^0 = \mathbf{v}^0 = 0$, $t = 0$;

While $\mathbf{x}$ not converged

$t = t + 1$

$\mathbf{g} = \nabla_{\overline{\mathbf{x}}}\mathcal{L}_{\text{Likelihood}}\left(\mathbf{x}^{t-1}\right) + \beta\left(\mathbf{x}^{t-1} - \mathcal{C}(\mathbf{x})\right)$

$\mathbf{u}^t = \gamma_1 \mathbf{u}^t + (1-\gamma_1)\mathbf{g}$

$\mathbf{v}^t = \gamma_2 \mathbf{v}^t + (1-\gamma_2)|\mathbf{g}|^2$

Bias correction

$\widehat{\mathbf{u}} = \frac{\mathbf{u}^t}{1-\gamma_1^t}$, $\widehat{\mathbf{v}} = \frac{\mathbf{v}^t}{1-\gamma_2^t}$

Update

$\mathbf{x}^t = \mathbf{x}^{t-1} - \frac{\eta}{\sqrt{\widehat{\mathbf{v}}}+\epsilon}\,\widehat{\mathbf{u}}$, or

$\mathbf{x}^t = \mathbf{x}^{t-1} - \frac{\eta}{\sqrt{\widehat{\mathbf{v}}}+\epsilon}\left[\gamma_1\,\widehat{\mathbf{u}} + (1-\gamma_1)\mathbf{g}\right]$ (NAdam)

Optimizer 2: Complex AdaBelief

Initialize $\mathbf{x}^0$, $\mathbf{u}^0 = \mathbf{s}^0 = 0$, $t = 0$;

While $\mathbf{x}$ not converged

$t = t + 1$

$\mathbf{g} = \nabla_{\overline{\mathbf{x}}}\mathcal{L}_{\text{Likelihood}}\left(\mathbf{x}^{t-1}\right) + \beta\left(\mathbf{x}^{t-1} - \mathcal{C}(\mathbf{x})\right)$

$\mathbf{u}^t = \gamma_1 \mathbf{u}^t + (1-\gamma_1)\mathbf{g}$

$\mathbf{s}^t = \gamma_2 \mathbf{s}^t + (1-\gamma_2)\left|\mathbf{u}^t - \mathbf{g}\right|^2$

Bias correction

$\widehat{\mathbf{u}} = \frac{\mathbf{u}^t}{1-\gamma_1^t}$, $\widehat{\mathbf{s}} = \frac{\mathbf{s}^t}{1-\gamma_2^t}$

Update

$\mathbf{x}^t = \mathbf{x}^{t-1} - \frac{\eta}{\sqrt{\widehat{\mathbf{s}}}+\epsilon}\,\widehat{\mathbf{u}}$

Optimizer 3: Complex Lion

Initialize $\mathbf{x}^0$, $\mathbf{u}^0 = \mathbf{v}^0 = 0$, $t = 0$;

While $\mathbf{x}$ not converged

$t = t + 1$

$\mathbf{g} = \nabla_{\overline{\mathbf{x}}}\mathcal{L}_{\text{Likelihood}}\left(\mathbf{x}^{t-1}\right) + \beta\left(\mathbf{x}^{t-1} - \mathcal{C}(\mathbf{x})\right)$

$\mathbf{v}^t = \gamma_1 \mathbf{u}^t + (1-\gamma_1)\mathbf{g}$

$\mathbf{u}^t = \gamma_2 \mathbf{u}^t + (1-\gamma_2)\mathbf{g}$

Update

$\mathbf{x}^t = \mathbf{x}^{t-1} - \frac{\eta}{|\mathbf{v}^t|+\epsilon}\mathbf{v}^t$

Note that we do not use the second-order gradient for the optimization mainly due to three points. First, the FD-PR loss function is complicated to calculate the second-order gradient. For large-scale input/out, computing the second-order gradient can be also time-and-spatial consuming. Second, the optimizers accelerate the gradient descent while do not significantly increase the computational complexity and memory requirement. The last and most important, the wavefront retrieval is very non-convex, meaning that using the second-order gradient can indeed speed up the optimization but cannot get rid of the local minimum. While the optimizers enable the engine to escape the local minimum, looking for another (probably better) local minimum as can be seen in Fig. S3 (a) and (b).

Note S8 On the scaling function $\mathcal{S}$

In multi-measurement, not all images among a single dataset are at the same intensity level, meaning that the learning efficiency is different among one dataset. Taking Fourier ptychographic microscopy (FPM) as an example, as shown in Fig. S4 (a1), the FPM collects a large amount of dark-field images in order to achieve image super-resolution. Those dark-field images have many dark pixels compared to images from bright fields. Images with brighter pixels normally provide larger gradients than that of images with dimer pixels, directly minimizing the likelihood loss function using intensity measurement can lead to worse recovery for high-frequency components as shown in Fig. S4 (d1). The learning efficiency for low-frequency areas is better than that of high-frequency areas as shown in the Fourier spectrum.

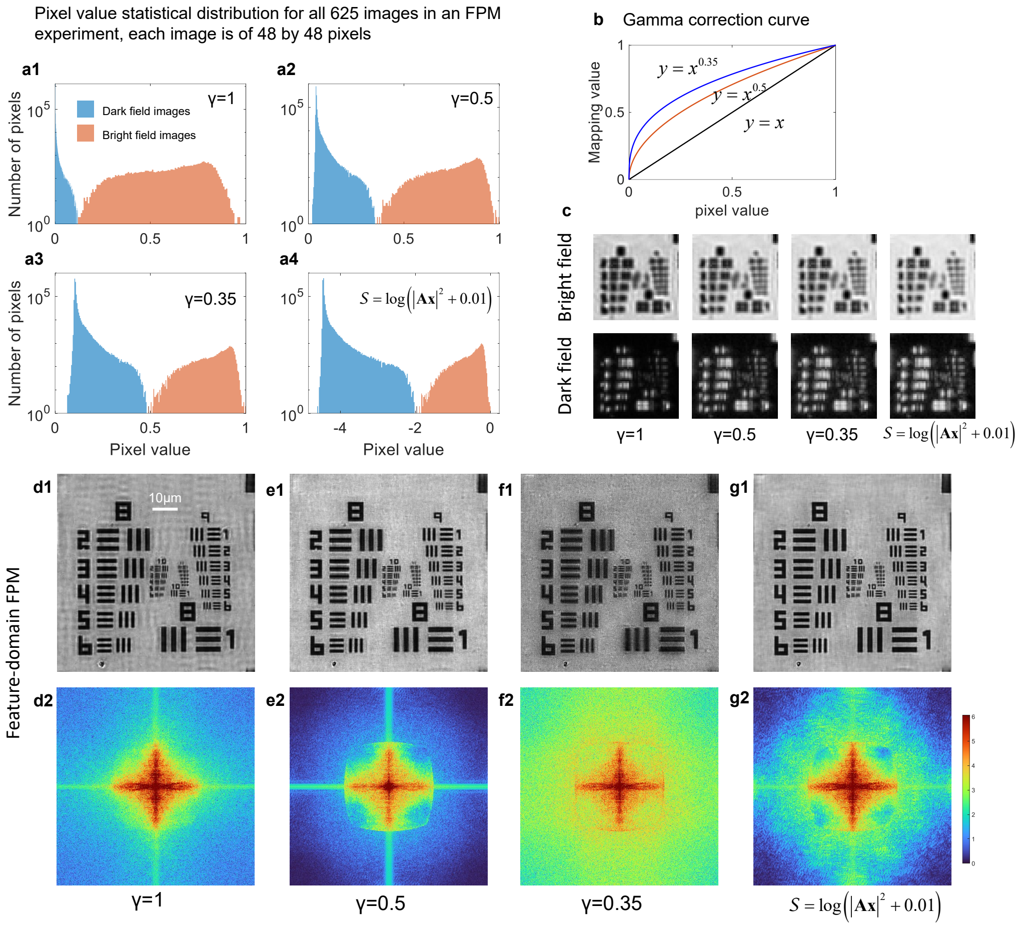

Fig. S4. Impact of function $\mathcal{S}$ on pixel distribution in a single dataset. The dataset is obtained from FPM experiments. (a1-a4) show pixel distribution with different $\mathcal{S}$. (b) is the gamma correction curve. (c) are the change of pixel value for a single image from bright- and dark-field. For FPM reconstruction using FD-PR, three values $\gamma = 1$, $\gamma = 0.5$ and $\gamma = 0.35$ were tested with results shown in (d1,d2), (e1,e2) and (f1,f2). (g1,g2) shows the reconstructions using the logarithm function as a replacement for gamma correction.

The scaling function, $\mathcal{S}$, provides mapping on the intensity measures. An example of function $\mathcal{S}$ is Gamma correction, where $\mathcal{S}(\mathbf{x}) = \mathbf{x}^{\gamma}$. As shown in the gamma correction curve in Fig. S4 (b), when $\gamma = 1$, it implies that the intensity was used to calculate the likelihood, while $\gamma = 0.5$ the amplitude was used which is widely implemented in phase retrieval. Nevertheless, $\mathcal{S}$ can be an arbitrary function that re-scales the dynamic range of input images. This will benefit wavefront recovery providing there are multiple measurement in a single dataset. The change of image intensity for collected images is shown in Fig. S4 (c).

With the scale function $\mathcal{S}$, the intensity distribution within a single dataset can be balanced given as Fig. S4 (a2-a3), benefiting the learning process and optimization. For example, when $\gamma = 0.5$ or $\gamma = 0.35$ as shown in Fig. S4 (e1) and Fig. S4 (f1), the dark-field images now contribute more compared with bright field images, thus the optimization obtains better results as shown in Fig. S4 (e2-f2). We also found that $\mathcal{S}$ can be in a more intricate form, say logarithm, results are shown in S4 (g1) and S4 (g2).

Note S9 Additional experimental results for FD-PR on FPM

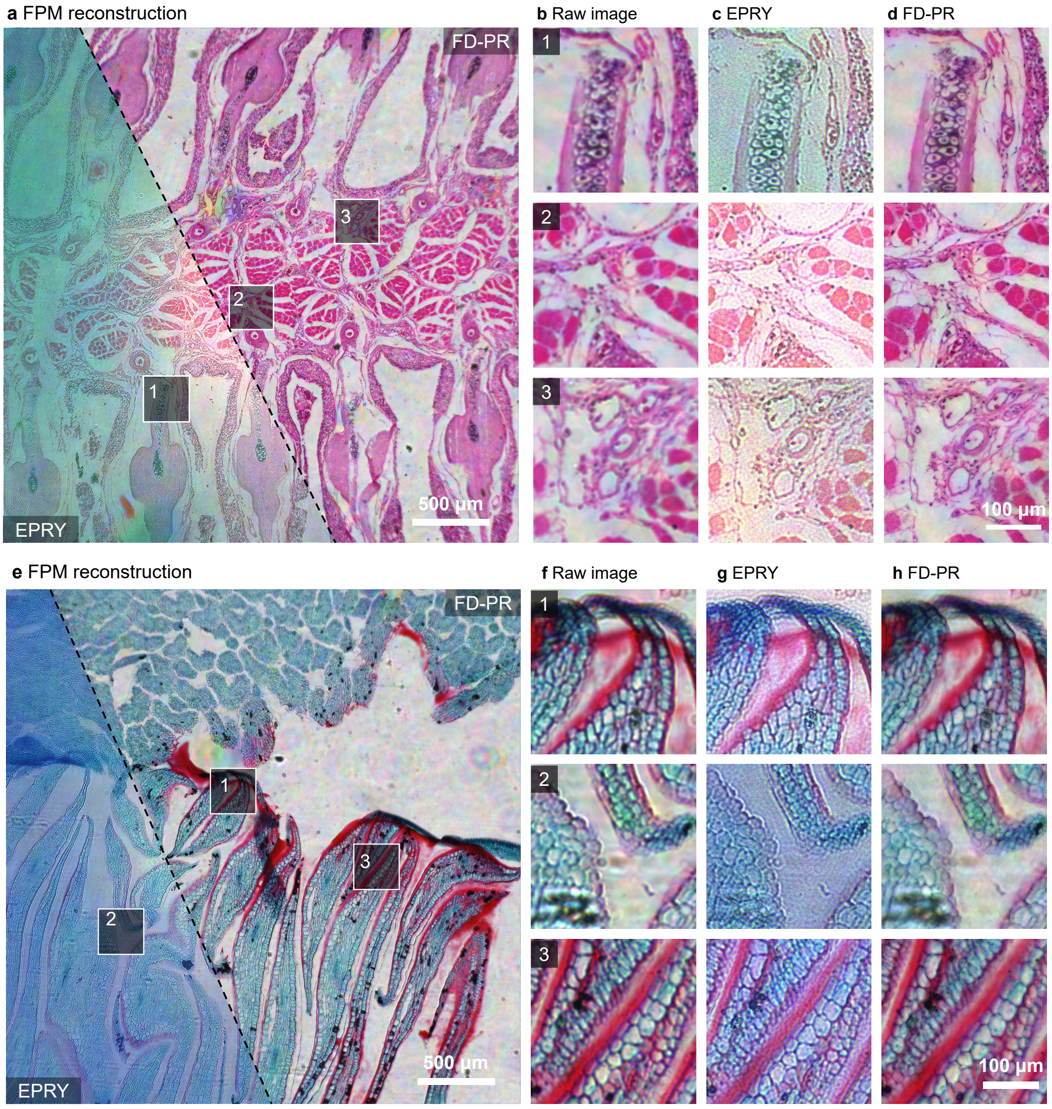

Fig. S5. (a) FPM reconstruction sample of a fish gill. (b, c, and d) zoomed in on the area in the black boxes. The gigapixel image is available on Gigapan. (e) FPM reconstruction sample of a fruit of Ficus carica. (f, g, and h) zoomed in on the area in the black boxes. The gigapixel image is available on Gigapan.

This section presents additional experimental results in Fig. S5 for FD-PR, demonstrated on Fourier ptychographic microscopy. Results are compared against the widely-adapted embedded pupil recovery (EPRY) methods [25]. A circular LEDs panel consisting of a total of 93 LEDs is utilized for providing angular varying illuminations. The panel has 6 LED rings, with differences in the radius of each ring set at 9 mm. The arrangement, from inner to outer rings, includes 1, 8, 12, 16, 24, and 32 LED dots,

respectively. The panel is placed 110 mm from the sample. The central wavelengths of red, green, and blue LEDs are 0.683 μm, 0.523 μm, and 0.488 μm. An objective lens with a magnification of ×4 and NA of 0.1 is employed. The size of the camera pixel is 6.5 μm.

References

1. Fienup, J. R. Reconstruction of an object from the modulus of its Fourier transform. *Optics Letters* **3,** 27–29 (1978).
2. Fienup, J. R. Phase retrieval algorithms: a comparison. *Applied Optics* **21,** 2758–2769 (1982).
3. Kreutz-Delgado, K. The complex gradient operator and the CR-calculus. *arXiv preprint arXiv:0906.4835* (2009).
4. Kingma, D. P. & Ba, J. Adam: A method for stochastic optimization. *arXiv preprint arXiv:1412.6980* (2014).
5. Zhuang, J. *et al.* Adabelief optimizer: Adapting stepsizes by the belief in observed gradients. *Advances in neural information processing systems* **33,** 18795–18806 (2020).
6. Chen, X. *et al.* Symbolic discovery of optimization algorithms. *Advances in Neural Information Processing Systems* **36** (2024).
7. Jiang, S. *et al.* Spatial-and Fourier-domain ptychography for high-throughput bio-imaging. *Nature Protocols* **18,** 2051–2083 (2023).
8. Knoll, F., Bredies, K., Pock, T. & Stollberger, R. Second order total generalized variation (TGV) for MRI. *Magnetic Resonance in Medicine* **65,** 480–491 (2011).
9. Wang, Y., Yang, J., Yin, W. & Zhang, Y. A new alternating minimization algorithm for total variation image reconstruction. *SIAM Journal on Imaging Sciences* **1,** 248–272 (2008).
10. Zhang, W. *et al.* Twin-Image-Free Holography: A Compressive Sensing Approach. *Physical Review Letters* **121,** 093902 (9 Aug. 2018).
11. Immerkaer, J. Fast noise variance estimation. *Computer vision and image understanding* **64,** 300–302 (1996).
12. Rizzi, A., Algeri, T., Medeghini, G. & Marini, D. *A proposal for contrast measure in digital images* in *Conference on colour in graphics, imaging, and vision* **2004** (2004), 187–192.
13. Romano, Y., Elad, M. & Milanfar, P. The little engine that could: Regularization by denoising (RED). *SIAM Journal on Imaging Sciences* **10,** 1804–1844 (2017).
14. Metzler, C., Schniter, P., Veeraraghavan, A. & Baraniuk, R. *prDeep: Robust Phase Retrieval with a Flexible Deep Network* in *Proceedings of the 35th International Conference on Machine Learning* (eds Dy, J. & Krause, A.) **80** (PMLR, 2018), 3501–3510.
15. Myung, I. J. Tutorial on maximum likelihood estimation. *Journal of mathematical Psychology* **47,** 90–100 (2003).
16. Pascal, F., Chitour, Y., Ovarlez, J.-P., Forster, P. & Larzabal, P. Covariance structure maximum-likelihood estimates in compound Gaussian noise: Existence and algorithm analysis. *IEEE Transactions on Signal Processing* **56,** 34–48 (2007).
17. Zhang, S. *et al.* FPM-WSI: Fourier ptychographic whole slide imaging via feature-domain backdiffraction. *Optica* **11** (2024).

18. Yang, J., Wright, J., Huang, T. S. & Ma, Y. Image super-resolution via sparse representation. *IEEE Transactions on Image Processing* **19,** 2861–2873 (2010).

19. Johnson, J., Alahi, A. & Fei-Fei, L. *Perceptual losses for real-time style transfer and super-resolution* in *Computer Vision–ECCV 2016: 14th European Conference, Amsterdam, The Netherlands, October 11-14, 2016, Proceedings, Part II 14* (2016), 694–711.

20. Zhao, H., Gallo, O., Frosio, I. & Kautz, J. Loss functions for image restoration with neural networks. *IEEE Transactions on Computational Imaging* **3,** 47–57 (2016).

21. Jain, P., Kar, P., *et al.* Non-convex optimization for machine learning. *Foundations and Trends® in Machine Learning* **10,** 142–363 (2017).

22. Rumelhart, D. E., Hinton, G. E. & Williams, R. J. Learning representations by back-propagating errors. *Nature* **323,** 533–536 (1986).

23. Dozat, T. Incorporating nesterov momentum into adam (2016).

24. Bernstein, J., Wang, Y.-X., Azizzadenesheli, K. & Anandkumar, A. *signSGD: Compressed optimisation for non-convex problems* in *International Conference on Machine Learning* (2018), 560–569.

25. Ou, X., Zheng, G. & Yang, C. Embedded pupil function recovery for Fourier ptychographic microscopy. *Opt. Express* **22,** 4960–4972 (Mar. 2014).
